# Hydrogen Peroxide Acts on Sensitive Mitochondrial Proteins to Induce Death of a Fungal Pathogen Revealed by Proteomic Analysis

**DOI:** 10.1371/journal.pone.0021945

**Published:** 2011-07-06

**Authors:** Guozheng Qin, Jia Liu, Baohua Cao, Boqiang Li, Shiping Tian

**Affiliations:** 1 Research Center for Molecular and Developmental Biology, Key Laboratory of Photosynthesis and Environmental Molecular Physiology, Institute of Botany, The Chinese Academy of Sciences, Beijing, China; 2 The Graduate University of the Chinese Academy of Sciences, Beijing, China; University of Wisconsin-Milwaukee, United States of America

## Abstract

How the host cells of plants and animals protect themselves against fungal invasion is a biologically interesting and economically important problem. Here we investigate the mechanistic process that leads to death of *Penicillium expansum*, a widespread phytopathogenic fungus, by identifying the cellular compounds affected by hydrogen peroxide (H_2_O_2_) that is frequently produced as a response of the host cells. We show that plasma membrane damage was not the main reason for H_2_O_2_-induced death of the fungal pathogen. Proteomic analysis of the changes of total cellular proteins in *P. expansum* showed that a large proportion of the differentially expressed proteins appeared to be of mitochondrial origin, implying that mitochondria may be involved in this process. We then performed mitochondrial sub-proteomic analysis to seek the H_2_O_2_-sensitive proteins in *P. expansum*. A set of mitochondrial proteins were identified, including respiratory chain complexes I and III, F_1_F_0_ ATP synthase, and mitochondrial phosphate carrier protein. The functions of several proteins were further investigated to determine their effects on the H_2_O_2_-induced fungal death. Through fluorescent co-localization and the use of specific inhibitor, we provide evidence that complex III of the mitochondrial respiratory chain contributes to ROS generation in fungal mitochondria under H_2_O_2_ stress. The undesirable accumulation of ROS caused oxidative damage of mitochondrial proteins and led to the collapse of mitochondrial membrane potential. Meanwhile, we demonstrate that ATP synthase is involved in the response of fungal pathogen to oxidative stress, because inhibition of ATP synthase by oligomycin decreases survival. Our data suggest that mitochondrial impairment due to functional alteration of oxidative stress-sensitive proteins is associated with fungal death caused by H_2_O_2_.

## Introduction

How the host cells in plants and animals respond to the invasion of fungi in a self-protective process is an important biological problem attracting a wide range of interests. The cellular environment within the host, such as constitutive and induced toxic molecules, represents a challenge to an invading fungus [1]. An oxidative burst (also known as the respiratory burst in phagocytes of mammals), during which large quantities of reactive oxygen species (ROS) are generated, is one of the earliest host responses after pathogen attack [2]–[4]. These ROS, mostly superoxide anion and hydrogen peroxide (H_2_O_2_), are produced by different host enzyme systems of plant or animal host cells. For instance, NADPH oxidases in the host plasma membrane catalyze the conversion of O_2_ to superoxide anions, which are converted to H_2_O_2_ either by spontaneous dismutation or by the catalytic activity of a cell wall superoxide dismutase [5]. The different types of ROS can not only destroy the invading pathogens directly [2], [6], [7] but also function as signaling molecules in the regulation of host defense response and programmed cell death (PCD), which restrict the spread of pathogens from the infection sites [8]–[10]. Although the critical roles of ROS in host PCD were revealed [11]–[14], the mechanisms by which ROS cause death in pathogenic fungi are poorly understood. Unraveling the mechanistic basis of death decisions in fungi is necessary for understanding of the important biological process of fungal invasion.

It was demonstrated that exogenous H_2_O_2_ at low fungicidal dose could induce PCD in the human pathogenic fungi, *Candida albicans* [7]. Many key markers of PCD were observed in *C. albicans*, accompanied by the elevated levels of intracellular ROS. This study indicated that H_2_O_2_ was able to translocate across the cytoplasmic membrane of *C. albicans* and react with the cellular components of the fungal pathogen. However, whether or not H_2_O_2_ triggers PCD through a specific intracellular component(s) is unknown. If there is such a component(s), the character of this component as well as the generation site and critical role of intracellular ROS has not been determined. Analysis of the antifungal peptide histatin 5 against *C. albicans* indicated that fungal mitochondria might contribute to the production of intracellular ROS [15]. The accumulation of ROS, which in turn led to respiration inhibition and loss of mitochondrial membrane potential, was the key element of the antifungal peptide induced killing mechanism in *C. albicans* [15]. These findings have pointed to a direct involvement of mitochondria in the process of death in fungal pathogens [16]. Mitochondria play critical roles in multiple cellular processes and have been extensively studied in various organisms such as yeasts [17], plants [18], and animals [19], [20], but little is known about this organelle in pathogenic fungi. Furthermore, although mitochondria have been shown to be sensitive to ROS, the specific site of inhibition of ROS in mitochondria remains largely unknown.

*Penicillium expansum* is a widespread fungal pathogen that causes decay in a variety of fruits, resulting in significant economic losses to the fruit industry. This strain is also of potential public health concern, because it produces toxic secondary metabolites, including patulin, citrinin, and chaetoglobosins [21]. The mycotoxin patulin has potential carcinogenic effects, and both Europe and the United States have established a maximum limit for patulin contamination in fruit-based products [22]. Thus, successful management of *P. expansum* becomes important for ensuring the quality and safety of various fruits. In previous research, we found that intracellular accumulation of ROS in *P. expansum*, caused by antifungal chemicals, was involved in cell damage and destruction, but the sensitive proteins for oxidative modification remain undetermined [23]. Here, we describe the molecular changes that lead to death in *P. expansum* in response to H_2_O_2_ stress, a situation being reported to restrict growth and spread of this pathogen during invasion [24], [25]. We found that exogenous H_2_O_2_ induced oxidative stress in the fungus and mitochondria might be sensitive to H_2_O_2_. In order to identify the ROS-sensitive proteins in mitochondria, we developed a procedure for high-purity mitochondrial separations from pathogenic fungi and, for the first time, examined the variations of the mitochondrial sub-proteome of a fungal pathogen under oxidative stress of H_2_O_2_. A set of mitochondrial proteins were identified including respiratory enzyme complexes, tricarboxylic acid cycle enzymes, and membrane carriers. Through characterization of protein functions, we demonstrate that mitochondrial impairment due to functional alteration of H_2_O_2_-sensitive proteins contributes to death of the fungal pathogen *P. expansum*.

## Results

### H_2_O_2_-Induced Oxidative Stress Causes Death of *P. expansum*

**Figure 1:**
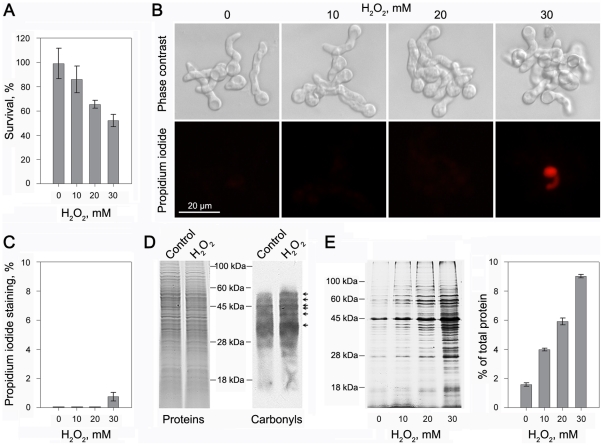
*P. expansum* exhibited a limited loss of plasma membrane integrity, but displayed protein oxidative damage. (A) H_2_O_2_ treatment decreases the percentage of fungal survival. The viability was assessed by survival plating assay, in which equal numbers of spores were plated onto solid media, and the numbers of formed colonies were counted the next day. (B) Propidium iodide (PI) staining indicates the limited loss of plasma membrane integrity. Before fluorescent staining, the fungal spores were cultured in potato dextrose broth medium until germination, and then treated with indicated concentrations of H_2_O_2_ for 60 min at 25°C. Scale bar, 20 µm. (C) Percentage of spores that lost plasma membrane integrity revealed by PI staining after treatment with H_2_O_2_. (D) H_2_O_2_ at 30 mM increases oxidative damage to total cellular proteins as indicated by immunodetection of protein carbonyl groups. The oxidatively modified proteins were separated by SDS-PAGE, blotted and detected using anti-dinitrophenyl-group antibodies (right panel). The arrowheads indicate protein bands that are highly carbonylated in spores under H_2_O_2_ stress. Coomassie Brilliant Blue staining was used to monitor equal loading of samples (left panel). (E) Detection of protein aggregation in *P. expansum* after H_2_O_2_ exposure. Aggregated proteins were isolated and analyzed by gel electrophoresis (left panel) as described in “Materials and Methods”. Quantification of aggregated proteins in relation to the total protein content is given in the right panel. Error bars represent standard deviation of three independent experiments.

H_2_O_2_ is reported to have a direct antimicrobial effect and be involved in defence reactions activated in plant tissues upon pathogen attack [2], [9]. To determine whether H_2_O_2_ inhibits survival of fungal pathogen *P. expansum*, the spores were exposed to H_2_O_2_ ranging from 0 to 30 mM. The viability of spores was assessed by survival plating assay [16], in which equal numbers of spores were plated onto potato dextrose agar (PDA) plates, and the numbers of formed colonies were determined. H_2_O_2_ caused a significant concentration-dependent loss of viability, as evidenced by the decreased number of formed colonies (Figure 1A). Approximately 50–60% decrease in viability was observed in spores upon exposure to 30 mM H_2_O_2_ for 60 min. This concentration was used in subsequent experiments. Viability was also evaluated by propidium iodide (PI) exclusion, a rapid and simple assay developed for the detection of dead and dying cells. PI is a fluorescent molecule that is membrane impermeant and generally excluded from viable cells. Only cells that have lost membrane integrity show red staining. As shown in Figure 1B, no spores were stained with PI after 60 min of exposure to H_2_O_2_ at concentrations lower than 30 mM. Most spores (more than 99%) were still not permeable to PI when the concentration of H_2_O_2_ reached 30 mM (Figure 1C), indicating that membrane damage was not the main reason for H_2_O_2_-induced fungal death.

To understand the mechanistic basis of fungal death, we initially examined H_2_O_2_-induced oxidative damage to cellular proteins by immunochemical detection of protein carbonyl groups. As indicated in Figure 1D, the intensity of immunostaining of several bands of cellular proteins increased dramatically after H_2_O_2_ exposure, suggesting that H_2_O_2_ exposure enhanced protein carbonylation levels. Because oxidatively damaged proteins tend to be denatured and aggregated, we then detected protein aggregation in spores that were treated with H_2_O_2_ for 60 min. Aggregated proteins were isolated and analysed by 12% SDS-PAGE followed by staining with Coomassie Brilliant Blue (CBB) R-250. Only a few aggregated protein bands were observed in the control, whereas significant amounts of aggregated proteins were induced after exposure to H_2_O_2_ (Figure 1E). The amount of aggregate proteins induced by H_2_O_2_ was concentration-dependent. Approximately 9% of total proteins aggregated in spores after treatment with 30 mM H_2_O_2_ (Figure 1E). These results show that the loss of fungal viability may be related to the changes of protein function induced by oxidative stress and that intracellular components might be involved in this process. In order to further elucidate the mechanisms by which H_2_O_2_ caused fungal death, we detected the H_2_O_2_-sensitive proteins in *P. expansum* using comparative proteomics analysis.

### Proteomic Analysis of Total Cellular Proteins Indicated that Mitochondria Might Be Sensitive to H_2_O_2_

**Figure 2:**
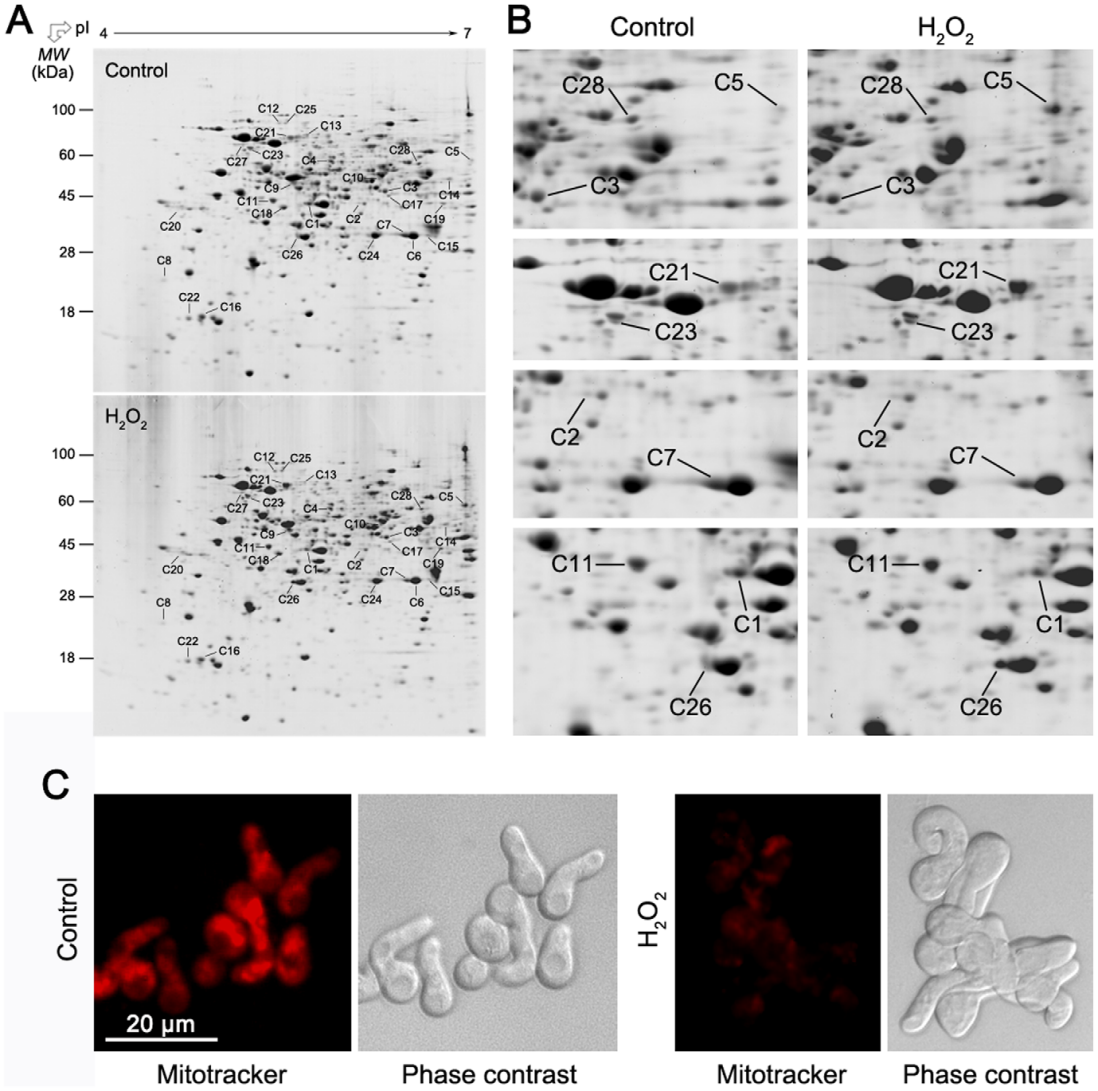
Proteomic analysis of total cellular proteins indicated that mitochondria might be sensitive to H_2_O_2_. (A) Differential expression of total cellular proteins in response to H_2_O_2_ in *P. expansum* visualized by 2D gel electrophoresis. The fungal spores were cultured in potato dextrose broth medium till germination, and then treated with 0 mM (control) or 30 mM H_2_O_2_ for 60 min at 25°C. Total cellular proteins were extracted from the fungi using a phenol-based method as described under “Materials and Methods”. Proteins (500 µg) were separated on 13 cm Immobiline Drystrip with a 4–7 linear pH gradient in the first dimension and visualized by Coomassie Blue staining. Numbers indicate proteins that were differentially expressed under H_2_O_2_ stress and subsequently identified by mass spectrometry (listed in Table S1). (B) Close-up views of putative mitochondrial proteins showing differential expression between control and H_2_O_2_-treated samples. (C) H_2_O_2_ induced collapse of mitochondrial membrane potential in the fungal pathogen as indicated by Mitotracker orange staining. The scale bar represents 20 µm.

The changes in expression profiles of total cellular proteins upon exposure to H_2_O_2_ were analyzed using high-resolution two-dimensional (2D) gel electrophoresis. To mimic the oxidative burst in hosts which is known to be triggered by penetrating hyphae, fungal spores were incubated in culture medium till germination (about 8.5 h), and then germlings were treated with 30 mM H_2_O_2_ for 60 min. In order to get rid of the contaminating compounds such as polysaccharides and lipids in the spores, proteins were extracted using a phenol-based method. Three independent samples of total cellular proteins isolated from H_2_O_2_-stressed and non-stressed spores were separated in the first dimension by isoelectric focusing on an immobilized linear pH gradient ranging from pH 4 to 7. These gel strips were then loaded onto SDS-PAGE gels and separated, producing the 2D IEF/PAGE gels (Figure 2A). More than 700 protein spots were reproducibly detected by Image Master 2D Elite software (GE Healthcare Bio-Sciences AB) on CBB-stained gels. Quantitative image analysis revealed a total of 28 protein spots that changed their intensities significantly (*p*>0.05) by more than 1.5-fold. Each of these spots was excised from the gels and in-gel digested with trypsin. Due to the lack of genome sequence information for this species, protein identification was performed with tandem mass spectrometry which yields MS/MS spectra. Table S1 summarizes the proteins that were identified by database searching in Mascot search engine. Because of the close phylogenetic relationship between *P. expansum* and *P. chrysogenum*, the source organism of almost all the identified proteins appeared to be *P. chrysogenum* whose genome sequence information was released recently [26]. The KEGG (Kyoto Encyclopedia of Genes and Genomes) database (http://www.genome.jp/kegg-bin/show_organism?org=pcs) was used to help define the protein function [27].

Interestingly we found that 10 identified spots (accounting for 37% of total identified spots) were likely to be of mitochondrial origin based on identifications and comparison to known mitochondrial proteins from model fungi (Figure 2B). To confirm their subcellular locations, the protein sequences were queried through three intracellular targeting prediction programs: TargetP (http://www.cbs.dtu.dk/services/TargetP/), Psort (http://psort.ims.u-tokyo.ac.jp/), and MitoProt (http://ihg2.helmholtz-muenchen.de/ihg/mitoprot.html) (Table S2). Among these 10 protein spots, seven are involved in tricarboxylic acid cycle and general metabolism, including malate dehydrogenase (Spot C26), ketol-acid reductoisomerase (Spot C3), acetyl-CoA C-acetyltransferase (Spot C5), pyruvate dehydrogenase E1 component, beta subunit (Spot C28), glycerol-3-phosphate dehydrogenase (Spot C1), mitochondrial 3-hydroxyisobutyryl-CoA hydrolase (Spot C7), and mitochondrial kynurenine aminotransferase (Spot 11). Their expressions were induced (Spots C26) or repressed (Spots C1, C3, C5, C7, C11 and C28) under H_2_O_2_ stress. Of the remaining proteins, two protein spots (Spots C21 and C23) were identified as the same protein, molecular chaperone DnaK whose expression was induced upon exposure to H_2_O_2_. One spot (Spot C2), which was likely a breakdown product based on the gel-based size of the protein spot, was identified as F-type H+-transporting ATPase subunit beta. The observation that mitochondrial proteins occupied a large proportion of the differentially expressed proteins indicates that mitochondria may play a crucial role in the survival of fungal pathogen under oxidative stress caused by H_2_O_2_. We then determined mitochondrial function by detecting mitochondrial membrane potential (ΔΨ_m_) using the fluorescent probe Mitotracker orange [17]. This probe irreversibly stains mitochondria in a ΔΨ_m_-dependent fashion, staining more brightly when ΔΨ_m_ is high. As shown in Figure 2C, ΔΨ_m_ decreased significantly after H_2_O_2_ exposure, indicating the resulting loss of mitochondrial membrane potential and hence likely of their function. This suggests their involvement in fungal death. To identify the proteins sensitive to ROS, the mitochondrial proteome variations under H_2_O_2_ stress were analyzed in the subsequent studies.

### Identification of H_2_O_2_-Sensitive Proteins in Fungal Mitochondria

The quality of a mitochondrial proteome is largely dependent on the purification of the isolated mitochondria away from other cellular contaminants. We have utilized a two-step Percoll gradient for separation of high-purity mitochondria from the fungi. On the first step-gradient, mitochondria formed a broad band at the 25%∶40% interface (Figure S1A). The mitochondrial band was taken out and further purified by a second self-forming gradient consisting of 28% Percoll. After centrifugation, mitochondria were enriched near the bottom of the gradient (Figure S1B). Mitochondrial proteins were separated by 2D gel electrophoresis using 13 cm Immobiline Drystrip with a pH 3–10 nonlinear gradient (Figure S2). To ensure reproducibility, the experiment was carried out three times independently, and the Student *t* test was carried out to determine the statistically significant alterations. Approximately 550 protein spots could be detected on 2D gels after ignoring very faint spots and spots with undefined shapes and areas. Image analysis revealed that 18 proteins were differentially expressed upon exposure to H_2_O_2_. These protein spots were excised from the gels, digested with trypsin, and subjected to tandem mass spectrometry. Among these protein spots, sixteen were successfully identified with Mowse scores greater than the threshold, which is 44 (Table S3). Their subcellular locations were analyzed by the intracellular targeting prediction programs: TargetP, Psort, and MitoProt (Table S4). Of the 16 protein spots identified, 11 were predicted to be mitochondrial by all three prediction programs. In the list of 5 proteins not assessed to be mitochondrial targeted by any programs, 4 are well-known mitochondrial proteins including NADH-ubiquinone oxidoreductase 12 kDa subunit (M16), NAD-dependent formate dehydrogenase (M2), mitochondrial import protein Metaxin (M10), and mitochondrial phosphate carrier protein (Mir1), putative (M9). The lack of prediction of these proteins may be explained by the absence of mitochondrial targeting presequences or of cryptic presequences recognized by these programs [28], [29]. Our results suggest that the two Percoll gradient density separations are efficient for high-purity mitochondria preparations.

The identified mitochondrial proteins can be grouped into particular biochemical groups. Several proteins involved in the electron transport were identified. Marked decreases in abundance were recorded in the two subunits of mitochondrial respiratory complex I (Spots M4 and M8), whereas an increase in abundance was observed in the 12-kDa subunit (Spot M16) during H_2_O_2_ stress. It is noticeable that the abundance of complex III core subunit 2 (Spot M3) was decreased more than two-fold upon exposure to H_2_O_2_. Among the spots identified as F_1_F_0_ ATP synthase subunits (alpha, beta, and gamma chains), all appeared to decrease in abundance during H_2_O_2_ stress, and several appeared to be a breakdown product rather than the intact proteins (Spots M6, M7, and M11). The function of these proteins in the survival of fungal pathogen under oxidative stress was subsequently studied.

### Mitochondrial Respiratory Complex III Contributes to the Production of ROS upon Exposure to H_2_O_2_

**Figure 3:**
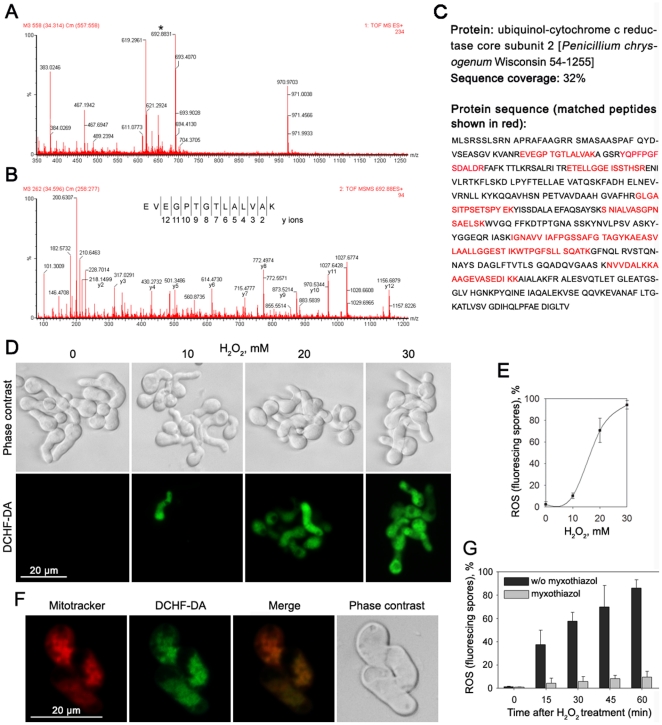
Involvement of respiratory complex III in ROS production in *P. expansum* upon exposure to H_2_O_2_. (A–C) Identification of complex III by mass spectrometry. Peptides were produced by in-gel tryptic digestion and analyzed by ESI-Q-TOF MS/MS. (A) MS spectra of the precursor ion. The ion with m/z value 692.88 marked with an asterisk was analyzed by MS/MS. (B) MS/MS spectra obtained from precursor ions 692.88. The y-ions and the corresponding peptide sequence are indicated. (C) Database search revealed this protein to be ubiquinol-cytochrome *c* reductase (complex III) core subunit 2. (D) Production of ROS in *P. expansum* after H_2_O_2_ treatment assessed by the oxidant-sensitive probe 2′,7′-dichlorodihydrofluorescein diacetate (DCHF-DA). Before fluorescent staining, the germinated spores were treated with the indicated concentrations of H_2_O_2_ for 60 min at 25°C. (E) Percentage of spores that contain ROS revealed by DCHF-DA staining after treatment with H_2_O_2_. Error bars represent standard deviation of three independent experiments. (F) Co-localization of the Mitotracker orange (a fluorescent dye that stains mitochondria) and DCHF-DA staining inside the germlings. (G) Effect of myxothiazol (2 µM), the complex III inhibitor, on H_2_O_2_-induced increase in ROS production. The scale bars represents 20 µm.

The mitochondrial electron-transfer chain is considered to be the major intracellular source of accidental ROS production [30], [31]. In animals and yeasts, the mitochondrial respiratory chain complex III has been reported to be required for ROS production induced by hypoxia [32], [33]. In the present study, it is notable that the respiratory complex III core subunit 2 decreased markedly in abundance under H_2_O_2_ stress. This protein was successfully identified by MS/MS analysis with high sequence coverage and multiple peptide matches (Figure 3A–C). The changes in the abundances of complex III under oxidative stress of H_2_O_2_ might cause the accumulation of ROS in the fungal pathogen *P. expansum*. We then detected the ROS levels in spores treated with or without H_2_O_2_ by the oxidant-sensitive probe 2′,7′-dichlorodihydrofluorescein diacetate (DCHF-DA). The results showed that, compared with the controls, more spores were stained with DCHF-DA under H_2_O_2_ stress, indicating that an increasing amount of ROS was produced in spores exposed to exogenous H_2_O_2_ (Figure 3D and 3E).

To verify that mitochondrial respiratory complex III is involved in ROS production in *P. expansum* under oxidative stress, we first detected the cellular location of ROS formation. The spores were double stained with DCHF-DA and Mitotracker orange, a fluorescent dye that stains mitochondria in a membrane potential-dependent manner. The co-localization of fluorescent signals of Mitotracker and DCHF-DA confirmed that mitochondria are involved in stress-induced ROS production (Figure 3F). Further investigation was conducted to determine that the accumulation of ROS observed in this study was complex III-linked. We tested the effects of myxothiazol, the inhibitor that interrupts the electron transfer within complex III [17]. Applying this inhibitor, we found a significant reduction in the production of ROS under H_2_O_2_ stress (Figure 3G). By comparison, the complex I inhibitor rotenone did not have this effect (data not shown). Taken together, these data suggest that the respiratory complex III is a major site of ROS production in *P. expansum*.

**Figure 4:**
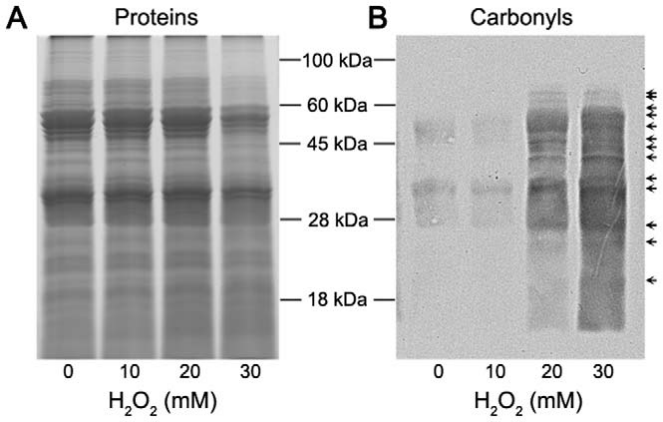
Oxidative damage of mitochondrial proteins in *P. expansum* under H_2_O_2_ stress. The germinated fungal spores were treated with the indicated concentrations of H_2_O_2_ for 60 min at 25°C and mitochondria were purified through a two-step Percoll density gradient centrifugation as described under “Materials and Methods”. The oxidatively modified mitochondrial proteins were separated by SDS-PAGE and detected using anti-dinitrophenyl-group antibodies. The arrowhead indicates protein bands that are highly carbonylated under H_2_O_2_ stress. (A) Protein staining with Coomassie Brilliant Blue to monitor equal loading of samples. (B) Immunodetection of carbonylated proteins.

Since ROS accumulation was shown to be associated with whole-cell protein oxidative damage (Figure 1), we investigated mitochondrial protein carbonylation using anti-DNP (anti-dinitrophenyl-group) antibodies. As shown in Figure 4B, several bands of mitochondrial proteins were selectively modified under H_2_O_2_ stress, especially when the concentration of H_2_O_2_ reached 20 mM. Basal levels of protein carbonyl content are caused by ROS generated through normal metabolic activity, such as mitochondrial respiration [34]. Protein carbonylation levels increased when the spores were exposed to higher concentration of H_2_O_2_. These proteins ranged in sizes with apparent molecular masses from 20 to 80 kDa. Our results suggest that special mitochondrial proteins are sensitive to oxidative damage.

### Time Course of ROS Generation and the Loss of Mitochondrial ΔΨ_m_

**Figure 5:**
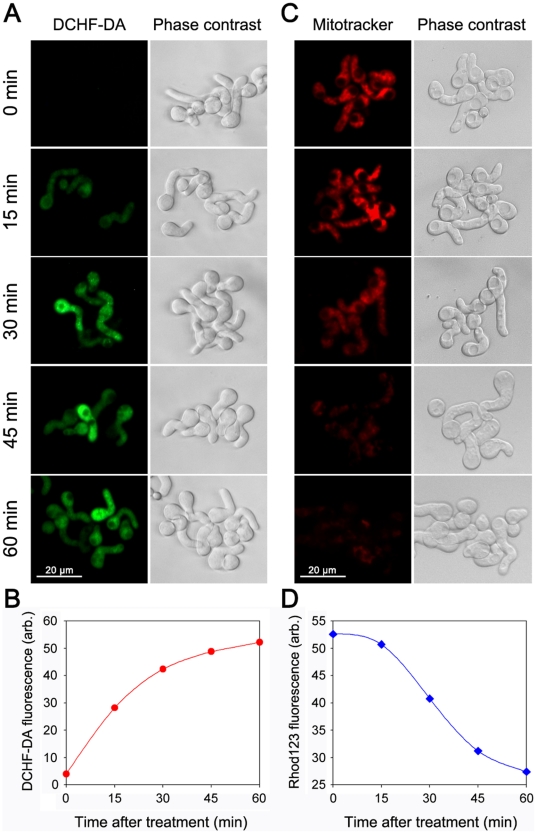
The time course of ROS generation and mitochondrial membrane potential changes after H_2_O_2_ exposure. The fungal spores were cultured in potato dextrose broth medium till germination, and then treated with 30 mM H_2_O_2_ at 25°C. The production of ROS was assessed using 2′,7′-dichlorodihydrofluorescein diacetate (DCHF-DA), at the time indicated, by microscopy (A) or by flow cytometry (B). H_2_O_2_-induced loss of mitochondrial membrane potential (ΔΨ_m_) were determined by microscopy with Mitotracker orange (C) or by flow cytometry using rhodamine 123 (Rhod123) (D). Fluorescence intensity of DCHF-DA or Rhod123, expressed in arbitrary units, is an average value calculated with at least 10,000 spores. The scale bars in (A) and (C) represents 20 µm.

It was suggested that the loss of ΔΨ_m_ was directly associated with the accumulation of ROS [35]. To test this hypothesis, we monitored the kinetic relationship between ROS production and ΔΨ_m_ collapse in the fungal pathogen *P. expansum*. The formation of ROS was determined with the oxidant-sensitive probe DCFH-DA by fluorescence microscopy and flow cytometry. It was shown that H_2_O_2_ caused a time-dependent change in ROS. The increase in DCFH-DA fluorescence intensity was evident 15 min after H_2_O_2_ was added, either by microscopy (Figure 5A) or by flow cytometric analysis (Figure 5B), indicating that ROS began to accumulate at this moment. By comparison, the changes in ΔΨ_m_, which was detected by microscopy with fluorescent probe Mitotracker orange (Figure 5C) or by flow cytometry with rhodamine 123 (Rhod123) (Figure 5D), was not evident until 30 min after the spores were treated with H_2_O_2_. These results indicate that ROS production preceded the loss in ΔΨ_m_ upon exposure to H_2_O_2_ in *P. expansum*. Our data agree with those reported by Herrera et al. [35], who concluded that ROS accumulation took place earlier than the ΔΨ_m_ loss after treatment with apoptosis-inducing factor in animal cells.

### Involvement of Intracellular ATP Generation and Mitochondrial ATP Synthase in H_2_O_2_-Induced Fungal Death

**Figure 6:**
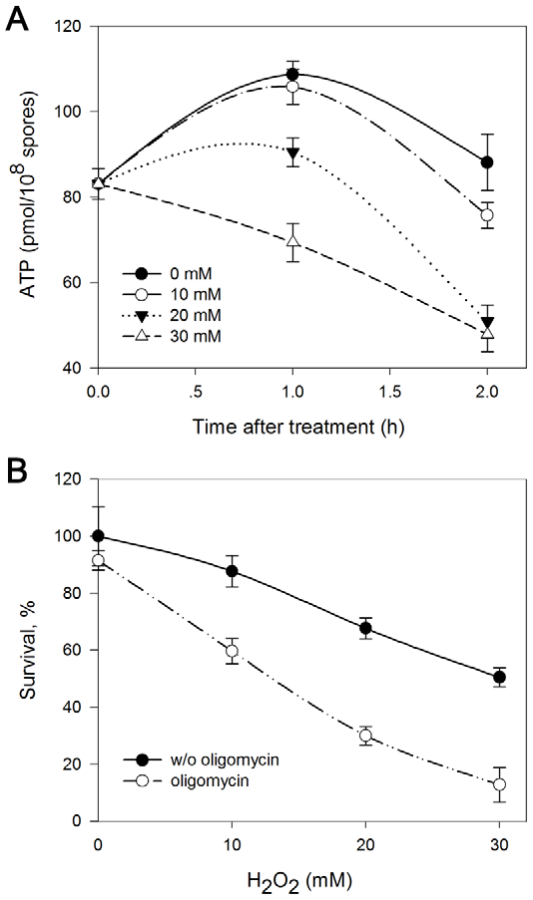
Involvement of mitochondrial ATP synthase in H_2_O_2_-induced fungal death. (A) A decline in the intracellular ATP levels occurred in spores exposed to H_2_O_2_. The fungal spores were cultured in potato dextrose broth medium till germination, and then treated with the indicated concentrations of H_2_O_2_ for 1 or 2 h at 25°C. ATP was measured as described under “Materials and Methods”. (B) Effect of oligomycin, the inhibitor of mitochondrial ATP synthase, on H_2_O_2_-induced fungal death. Spores were pretreated for 1 h with or without 2 µM oligomycin, followed by 1 h incubation with the indicated concentrations of H_2_O_2_. The viability was assessed by plating equal numbers of spores onto solid media and determining the numbers of formed colonies the next day. Error bars represent standard deviation of three independent experiments.

ATP synthase is the crucial enzyme for ATP generation in mitochondria. Since reduced amounts and degradation of several subunits of mitochondrial ATP synthase complex were observed in the mitochondrial sub-proteomic analysis, we measured the changes in ATP production in the fungal pathogen under H_2_O_2_ stress. Figure 6A show that exposure of spores to H_2_O_2_ caused a concentration-dependent decrease in the intracellular level of ATP. The loss of ATP appeared to be rapid and significant, occurring after 60 min of exposure to 20 mM H_2_O_2_. Increasing the concentration of H_2_O_2_ substantially increased ATP depletion. With the increase of exposure time, the ATP levels decreased among all treatments.

A decline in mitochondrial ATP generation can decrease the efficiency of energy-dependent processes such as cell defenses against environmental stress [36]. We then measured the relationship between ATP production and H_2_O_2_-induced fungal death by using the inhibitor of ATP synthase, oligomycin, which decreased the intracellular level of free ATP. Exposure of *P. expansum* to 10, 20, and 30 mM H_2_O_2_ for 60 min led to 12%, 32%, and 50% losses in viability, respectively (Figure 6B). By comparison, pretreatment of the germlings with 2 µM oligomycin before exposure to H_2_O_2_ at 10, 20, and 30 mM led to greater losses in fungal viability, that is 40%, 70%, and 87%, respectively. It was shown that, at the concentrations employed (2 µM), oligomycin was not cytotoxic itself in the survival plating assay. Taken together, these results suggest that decline of intracellular ATP levels was associated with fungal death caused by H_2_O_2_ stress and the change in the function of mitochondrial ATP synthase was involved in this process.

## Discussion

Upon recognition of a microbial pathogen, a plant or an animal host starts to release high levels of ROS, mostly superoxide anion and H_2_O_2_ [37], [38]. This rapid production of ROS, called the oxidative burst, would provide an extremely hostile environment for pathogens [39]. Here we show that H_2_O_2_ exposure caused a concentration-dependent loss of viability in the phytopathogenic fungus *P. expansum* (Figure 1A). To investigate the mechanisms whereby H_2_O_2_ caused fungal death, we firstly detected the integrity of the plasma membrane. Damage to the plasma membrane can result in loss of osmotic balance and influx of fluids and ions, as well as loss of proteins and ribonucleic acids, eventually leading to the onset of cell death [40]–[42]. It is well recognized that oxygen radicals can rapidly lead to disintegration of biological membranes, resulting in cell death. However, in the present study, the loss of plasma membrane integrity was not evident in *P. expansum* upon exposure to half lethal dose of H_2_O_2_. This indicated that membrane damage was not the main reason for H_2_O_2_-induced fungal death. We then examined intracellular changes in *P. expansum* that accompanied fungal death after exposure to H_2_O_2_. An increase in the level of protein oxidation and aggregation was observed in whole-cell lysates, indicating that protein oxidative modification may be associated with H_2_O_2_-induced fungal death. To elucidate the detailed mechanisms by which H_2_O_2_ reduces fungal viability, we performed comparative proteomics analysis of the total cellular proteins. Among the identified proteins, the most notable were those located in mitochondria, which occupied a large proportion of the total identified spots. This observation indicates that the mitochondrial proteins seem disproportionally affected under oxidative stress of H_2_O_2_ and that their inactivation might be contributing to fungal death.

In the present study, death of *P. expansum* was accompanied by a severe decrease of ΔΨ_m_, a crucial parameter of mitochondrial function, suggesting that mitochondria were impaired upon exposure to exogenous H_2_O_2_. Mitochondria are vital organelles within the eukaryotic cell that perform a variety of biochemical functions [43]. The most prominent roles of mitochondria are to produce ATP though the process of oxidative phosphorylation, and to regulate cellular metabolism [44]. In addition to supplying cellular energy, mitochondria play a critical role in a range of other processes, such as signaling, cellular differentiation, cell death, as well as the control of the cell cycle and cell growth [45]. In recent years, mitochondria have been identified as important organelles for the infection process of fungal pathogens. Ingavale et al. [46] demonstrated that mitochondria were necessary for the environmental fungal pathogen *Cryptococcus neoformans* to survive in low oxygen conditions in the host environment during infection. The authors found that mutants harboring mutations in the genes related to mitochondrial functions had mitochondrial membrane permeability defects and lowered respiration rates and were more sensitive to low oxygen conditions. Moreover, it was reported that the mitochondrial Mrb1 protein in *Ustilago maydis* [47] and Fow1 protein in *Fusarium oxysporum* [48] were specifically required for pathogenicity of these two phytopathogenic fungi.

We present here for the first time a comparative analysis of the mitochondrial sub-proteome of a fungal pathogen to seek the H_2_O_2_-sensitive proteins in mitochondria. Sub-proteomic analyses enable the study of protein expression localized to a particular organelle, thereby providing additional insight into the protein function in a given physiological state of the cell [49]. Mitochondrial sub-proteomic analysis depends largely on the purification of these organelles away from other cellular contaminants. We developed a protocol to obtain high-purity mitochondria from this fungal pathogen through differential centrifugation, followed by separation over two Percoll gradients. Mitochondrial proteins of *P. expansum* were isolated and separated by 2D gel electrophoresis. A set of 16 mitochondrial proteins that appeared to change significantly in abundance in response to H_2_O_2_ were identified by MS/MS analysis (Table S3). Fifteen of the 16 proteins were located in the mitochondria based on the intracellular targeting prediction programs (TargetP, Psort, and MitoProt) and comparison with known of the mitochondrial proteins in fungi. Notably, subunits of mitochondrial respiratory chain including complex I, complex III, and complex V (F_1_F_0_ ATP synthase) were identified. Particular attention was paid to the respiratory complex III, because this protein was shown to contribute to hypoxia-induced ROS production in animals and yeasts [32], [33]. The identification of complex III made us hypothesize that exogenous H_2_O_2_ might cause the accumulation of ROS within the fungus. To verify this hypothesis, we monitored the intracellular ROS levels by using DCHF-DA, a cell-permeable ROS indicator that penetrates live cells but does not fluoresce unless oxidized by ROS [50]. As expected, more spores were stained with DCHF-DA under H_2_O_2_ stress, implying that more ROS were generated under this condition (Figure 3D and 3E). Possible sources of cellular ROS include leakage from the mitochondrial electron transport chain as well as a number of ROS-generating plasma membrane and cytosolic enzymes [51]. In this study, the fluorescent signals of DCHF-DA could be co-localized with those of Mitotracker, a fluorescent dye that stains mitochondria, indicating that mitochondria are responsible for H_2_O_2_-induced ROS production (Figure 3F). The decrease in the abundance of complex III core subunit 2, a subunit required for the assembly of the complex, might cause a disturbance of the respiratory chain, and thus lead to ROS generation. To evaluate whether mitochondrial complex III is a major source of ROS production upon exposure to H_2_O_2_, we assessed the effects of myxothiazol, the inhibitor of respiratory chain complex III [17]. Myxothiazol inhibits mitochondrial respiration because it obstructs the binding of ubiquinol at the Qo site of the complex, and therefore prevents electron transfer to the Rieske iron–sulphur protein and generation of ubisemiquinone at either Qo or Qi. We found that the percentage of spores containing ROS increased gradually after treatment with H_2_O_2_ in the control (without myxothiazol). However, when the spores were pretreated with myxothiazol, ROS production was significantly reduced (Figure 3G). The ROS-positive spores persisted at a low proportion after myxothiazol application throughout the whole period. These data suggest that the mitochondrial complex III serves as the major site for ROS production in *P. expansum* under the oxidative stress of H_2_O_2_.

Undesirable accumulation of ROS can cause oxidative injury to biological macromolecules. Being adjacent to the site of ROS generation, mitochondrial components such as proteins are particularly vulnerable to oxidative damage [52], [53]. We found that, concomitant with the enhanced generation of ROS, more oxidatively damaged proteins were detected in mitochondria as indicated by immunoblot detection. Protein oxidation can significantly affect biochemical characteristics such as enzyme activities, structural functions, and susceptibility to proteolysis [54], [55]. It has been reported that oxidative modification of mitochondrial proteins such as voltage-dependent anion-selective channel could result in the opening of the mitochondrial permeability transition pore, which ultimately caused the loss of ΔΨ_m_. By using a fluorescent probe, Mitotracker orange, we observed a significant decrease in ΔΨ_m_ under oxidative stress of H_2_O_2_. Further analysis of a causal link between ROS generation and ΔΨ_m_ changes showed that ROS production preceded the loss in ΔΨ_m_. Our data are consistent with the studies conducted in other organisms showing that the enhanced ROS generation is sufficient to induce the opening of mitochondrial megachannels, leading to the disruption of the ΔΨ_m_ [17], [35], [56]. Maintenance of ΔΨ_m_ has been reported to be associated with many mitochondrial functions, especially ATP generation, as it reflects the pumping of hydrogen ions across the inner membrane during the process of electron transport and oxidative phosphorylation, the driving force behind ATP production [57]. The collapse of ΔΨ_m_ found in this study suggests that ROS accumulation caused by exogenous H_2_O_2_ might reduce ATP production in the mitochondria.

Temporary or sustained loss of mitochondrial ATP synthesis has a major impact on the fidelity of cellular defenses and repair processes, which are energy-dependent [36]. Besides the loss of ΔΨ_m_, which may lead to the decrease of ATP levels, we observed reduced levels and degradation of several subunits of mitochondrial ATP synthase complex. ATP synthase is the last enzyme complex (complex V) of the respiratory enzyme complexes located in the mitochondrial inner membrane [58]. Under normal physiological conditions, ATP synthase generates ATP from ADP using the proton gradient created by the electron transport chain [59]. The alterations in ATP synthase are likely to have a significant effect on oxidative phosphorylation capacity, thereby limiting the synthesis of ATP. To determine whether the changes in ATP synthase influence ATP generation, we detected the intracellular levels of ATP. As shown in Figure 6A, ATP levels increased initially at the low concentrations of H_2_O_2_. This may represent the physiological response of the fungi to oxidative stress. However, when the concentration of H_2_O_2_ exceeded the ability to respond effectively, the fungi were killed and ATP levels declined quickly. It has been reported that depletion of cellular ATP made the cells more susceptible to oxidative stress in human fibroblasts [36]. We then analyzed the relationship between ATP synthesis and fungal survival under oxidative stress of H_2_O_2_ in the fungal pathogen. We found that reduction of intracellular levels of free ATP by oligomycin, the inhibitor of mitochondrial ATP synthase, could decrease viability of the fungus under oxidative stress. Taken together, our data suggest that mitochondrial ATP production is involved in the response of *P. expansum* to H_2_O_2_ stress. ATP synthase, as one of the H_2_O_2_-sensitive mitochondrial proteins, plays crucial role in this process.

In conclusion, we have shown that H_2_O_2_ has an effect on the viability of mitochondria and might lead to death in the fungal pathogen *P. expansum*. A procedure was developed for the isolation of highly pure mitochondria from this fungal pathogen and the changes in mitochondrial protein levels upon exposure to H_2_O_2_ were determined. Inhibitor studies of specific mitochondrial proteins suggest that the mitochondrial complex III contributes to the rapid generation of ROS in the mitochondria of the fungal pathogen, which in turn propagates mitochondrial protein oxidation and leads to loss of ΔΨ_m_. Moreover, we demonstrate that ATP synthase appears to be involved in the response of a fungus to oxidative stress of H_2_O_2_. The identification of mitochondrial proteins sensitive to H_2_O_2_ could provide a basis for future development of novel antifungal agents.

## Materials and Methods

### Fungal Strain, Growth Conditions, and Sensitivity to H_2_O_2_ Stress

The fungal pathogen, *Penicillium expansum* Link (CGMCC3.3703), was routinely grown on potato dextrose agar (PDA) plates for 14 days at 25°C. Spores were obtained by adding sterile distilled water containing 0.05% (v/v) Tween 80 to the surface of the culture and gently scraping with a sterile spatula. Spore suspensions were filtered through four layers of sterile cheesecloth to remove any hyphal fragments [60]. The survival plating assay was performed using the modified method of Helmerhorst et al. [16]. Aliquots of a spore suspension of *P. expansum* were added to wells of a 24-well microtitration plate containing potato dextrose broth (PDB) medium to obtain a final concentration of 1×10^6^ spores/ml. The microtitration plate was incubated at 25°C on a rotary shaker at 100 rpm till the spores germinated (about 8.5 h), and then H_2_O_2_ was added. After 60 min of incubation, an aliquot of the strains (50 µl) was spread on a 9 cm PDA plate and the colonies were counted the next day. The percentage of fungal survival was calculated based on counting the spores in the aliquot under the microscope before plating.

### Preparation of Total Cellular Proteins

Spores of *P. expansum* (1×10^6^ spores/ml) were cultured at 25°C in PDB medium. After the spores began to germinate, H_2_O_2_ was added at the final concentration of 30 mM, which led to approximately 50–60% decrease in fungal viability based on the survival plating assay as described above. The spores were collected after 60 min of incubation and washed thoroughly with phosphate buffered saline (PBS, pH 7.4) to remove residues. The total cellular proteins were extracted following the method of Li et al. [61]. Briefly, spores were frozen and powdered in liquid nitrogen with a pestle and mortar, followed by sonication (with cooling on ice) with 1 ml of extraction buffer containing 0.5 M Tris-HCl (pH 8.3), 2% (v/v) Nonidet P-40, 20 mM MgCl_2_, 2% (v/v) β-mercaptoethanol, and 1 mM PMSF. The fungal debris was removed by centrifugation (25,000×*g*, 20 min; 4°C) and the supernatant was mixed with an equal volume of Tris-HCl (pH 7.8) buffered phenol. The mixture was homogenized for 30 min at 4°C and finally centrifuged at 10,000×*g* for 30 min. The proteins in the phenol phase were precipitated with five volumes of saturated ammonium acetate in methanol overnight at −20°C and finally pelleted by centrifugation at 15,000×*g* for 30 min at 4°C. The protein pellets were washed twice with ice-cold saturated ammonium acetate in methanol followed by multiple ice-cold acetone washes. Proteins were air-dried and stored at −80°C until use.

### Mitochondria Purification and Protein Isolation

Fungal mitochondria were purified as described by Fečíková et al. [62] and Groebe et al. [63], with some modifications. All steps were performed at 4°C. Spores were frozen and powdered in liquid nitrogen with a pestle and mortar and suspended in three-fold volume of ice-cold extraction buffer containing 250 mM sucrose, 1 mM EDTA, 0.5% (w/v) polyvinylpyrrolidone-40, 10 mM *β*-mercaptoethanol, and 50 mM Tris-HCl, pH 7.2. The mixture was extensively homogenized for 30 min on ice in a 2°C cold room, and then the homogenate was centrifuged for 15 min at 1,200×*g*. The supernatants were decanted and centrifuged for 20 min at 17,000×*g*. The pellets were resuspended in wash buffer (250 mM sucrose, 50 mM Tris-HCl, pH 7.2) with a soft and small brush. The two centrifugation steps were repeated to reduce contamination from other organelles and the resultant pellets were resuspended in a small volume of wash medium as crude mitochondrial fraction. Mitochondria were subsequently purified by layering on top of a step Percoll gradient at 18, 25, and 40% (1∶4∶2) in wash buffer. After centrifugation for 45 min at 40,000×*g*, the mitochondria bands were aspirated and washed with wash medium by centrifugation at 15,000×*g* for 15 min. To further purify the mitochondria, concentrated mitochondria were loaded on top of a self-generated 28% (v/v) Percoll gradient and centrifuged for 30 min at 40,000×*g*. The mitochondrial layer was taken out and washed twice with wash buffer to remove Percoll. The purified mitochondria were finally resuspended in a small volume of wash medium and stored at −80°C.

For mitochondrial protein isolation, mitochondria were lysed by sonication on ice in lysis buffer containing 50 mM Tris-HCl (pH 7.2), 2% (v/v) *β*-mercaptoethanol, and 1 mM PMSF. The homogenate was centrifuged at 25,000×*g* for 20 min at 4°C. Proteins in the supernatant were precipitated for 30 min at 4°C with ice-cold TCA at a final concentration of 10% (w/v). The protein pellets were obtained by centrifugation at 25,000×*g* for 20 min at 4°C and washed three times with cold acetone to remove remaining TCA. Proteins were air-dried and stored at −80°C until use.

### Two-dimensional Gel Electrophoresis and Image Analysis

The proteins were solubilized in IEF buffer containing 7 M urea, 2 M thiourea, 4% (w/v) CHAPS, 1% (w/v) dithiothreitol (DTT), and 2% (v/v) carrier ampholytes (pH 3–10) before two-dimensional (2D) gel electrophoresis. Protein concentrations were quantified by the method of Bradford [64] using bovine serum albumin as a standard. Aliquots of 500 µg of total cellular or mitochondrial proteins resolved in 250 µl IEF buffer plus 0.001% (w/v) bromphenol blue were applied to rehydrate immobilized pH gradient gel strips (13 cm, pH 4–7 linear or pH 3–10 nonlinear; GE Healthcare Bio-Sciences AB, Uppsala, Sweden) for 16 h. The first-dimensional IEF was performed at 20°C for a total of 20 kVh on an Ettan IPGphor unit (GE Healthcare Bio-Sciences AB) following the manufacturer's instruction. After focusing, the proteins were reduced by incubating the gel strips with 1% w/v DTT for 15 min and alkylated with 4% w/v iodoacetamide for 15 min in 10 ml of equilibration buffer consisting of 150 mM Tris-HCl, pH 6.8, 8 M urea, 20% (v/v) glycerol, 2% (w/v) SDS. The strips were then transferred to 15% SDS-PAGE gels for the second-dimensional electrophoresis at a constant 30 mA per gel, using a SDS electrophoresis buffer containing 25 mM Tris (pH 8.3), 195 mM glycine, and 0.1% (w/v) SDS. Proteins in the gel were stained with Coomassie Brilliant Blue (CBB) R-250 solution containing 50% (v/v) methanol, 15% (v/v) acetic acid and 0.1% (w/v) CBB R-250.

The gels were scanned by a flatbed scanner (GE Healthcare Bio-Sciences AB) and filed in TIF format. Comparison of protein expression between samples in 2D gel images was performed using Image Master 2D Elite software (GE Healthcare Bio-Sciences AB). Three biological repeats resulting from independent experiments were used for each sample to account for experimental variation. The relative amount of a protein spot was calculated on basis of the spot volume. To reflect the quantitative variations in intensity of protein spots between different samples, the spot volume was normalized as a percentage of the total volume of all spots on the gel. The minimum requirement for spot quantification was spot presence in three biological repeats. Statistical analysis of the data was performed using SPSS software (SPSS Inc., Chicago, IL, U.S.A.). The normalized intensity of spots on three replicate 2D gels was averaged and a two-tailed nonpaired Student's *t*-test was used to determine whether the relative change was statistically significant between samples. Protein spots whose expression levels changed significantly were excised for protein identification.

### In-gel Digestion, Mass Spectrometry, and Database Searching

In-gel digestion was carried out as we previously reported with some modifications [23]. In brief, protein spots were manually excised from the gels and destained with 50 mM NH_4_HCO_3_ in 50% (v/v) methanol for 1 h at 40°C. The gels were completely dried in a vacuum centrifuge, and then enzymatically digested at 37°C for 16 h with 10 ng µl^−1^ trypsin. Digested peptides were extracted by three changes of 0.1% TFA in 50% acetonitrile, lyophilized and submitted to ESI-Q-TOF or MALDI-TOF/TOF tandem mass spectrometry.

ESI-Q-TOF MS/MS was performed with a Q-TOF mass spectrometer (Q-TOF-2; Micromass, Altrincham, U.K.), equipped with a z-spray source as described previously [23]. Before loading the digested peptide, the samples were desalted with ZipTipC_18_ (Millipore, Bedford, MA, U.S.A.), and eluted into 2 µl of 0.1% TFA in 50% acetonitrile. The instrument was externally calibrated using the fragmentation spectrum of the doubly charged 1571.68 Da (785.84 *m/z*) ion of fibrinopeptide B. The peptide samples were loaded into borosilicate nanoflow tip (Micromass) and the applied spray voltage was set to 800 V, with a sample cone working on 30 V. Dependent on the mass and charge state of the peptides, the collision energy was varied from 14 to 40 V. Peptide precursor ions were acquired over the m/z range 400–1900 Da in TOF-MS mode. Multiply charged (2+ and 3+) ions rising above the predefined threshold intensity were automatically selected for MS/MS analysis, and product ion spectra collected from m/z 50 to 2000. Peak lists were created by ProteinLynx (version 4.0; Micromass) with default parameter.

For MALDI-TOF/TOF MS/MS analysis, the peptides were resuspended with 5 mg/ml matrix solution (α-cyano-4-hydroxycinnamic acid in 50% acetonitrile containing 0.1% TFA) and spotted onto the MALDI target plates. Mass spectra were acquired on a MALDI-TOF/TOF mass spectrometer (4800 Proteomics Analyzer, Applied Biosystems, Framingham, MA, U.S.A.). The instrument was operated in the positive reflection mode and externally calibrated using the tryptic peptide mixtures of myoglobin (Sigma-Aldrich). MS spectra were acquired with 1600 laser shots per spectrum, whereas MS/MS spectra were obtained using 2500 laser shots per fragmentation spectrum. To acquire the MS/MS fragmentation spectra, the 10 strongest peaks of each MS spectra were selected as precursor ions, which exclude trypsin autolytic peptides and other known background ions. The 4000 Series Explorer™ software (Applied Biosystems) was used for spectra analyses and generation of peak list files, with parameters of a signal-to-noise threshold of 10 and a minimum area of 100.

For database searching, the generated peak lists were uploaded to Mascot MS/MS Ions Search program (version 2.1) on the Matrix Science (London, U.K.) public web site (http://www.matrixscience.com) and searched against NCBInr protein databases (version 20100403; 10810288 sequences and 3686216991 residues) with a taxonomy restriction to ‘Fungi’. Trypsin was chosen as the proteolytic enzyme, and one missed cleavage was permitted. Carbamidomethylation of cysteine and oxidation of methionine were selected as fixed modification and variable modification, respectively. Precursor and fragment ion mass tolerances were set to 0.5 and 0.6 Da for ESI-Q-TOF MS/MS and 0.2 and 0.3 Da for MALDI-TOF/TOF MS/MS, respectively. A total of 674158 sequences in the database were actually searched. Mascot uses a probability based “Mowse Score” to evaluate data obtained from tandem mass spectra. Mowse scores were reported as −10×log_10_(*p*) where *p* is the probability that the observed match between the experimental data and the database sequence is a random event. The best match is the one with the highest score. The matched peptides and individual peptide scores of the identified proteins are given in Table S5. Protein identification was based on at least three distinct peptides. If proteins were identified with only one matching peptide or by multiple peptides with each ion scored below the threshold, nearly complete Y-ion series and partial complementary B-ion series were present as determined by manual inspection (Figure S3). To eliminate the redundancy of proteins that appeared in the database under different names and accession numbers, the protein member belonging to the genus *Penicillium* or else with the highest protein score (top rank) was selected from the multi-protein family.

### Assay for Cytoplasmic Membrane Integrity, ROS Production, and Mitochondrial Membrane Potential

Integrity of cytoplasmic membrane was determined by fluorescence microscopy using 10 µg/ml propidium iodide (PI; Sigma-Aldrich), a fluorescent molecule that is commonly used for identifying membrane integrity of cells [65]. The oxidant-sensitive probe 2′,7′-dichlorodihydrofluorescein diacetate (10 µM; DCHF-DA; Sigma-Aldrich) was used to detect the intracellular ROS levels [17], [23]. Mitochondrial membrane potential was analyzed with Mitotracker orange (0.1 µg/ml; Molecular Probes) [17]. Spores were stained with different dyes for 10 min at 30°C, washed twice with PBS, and examined under a Zeiss Axioskop microscope (Carl Zeiss, Oberkochen, Germany). Images were collected using an Axiocam MRc digital camera (Carl Zeiss). ROS or mitochondrial membrane potential were also measured by flow cytometry using 10 µM DCHF-DA or 1 µg/ml rhodamine 123 (Rhod123) [19], respectively. Fluorescence was recorded on FL-1 channel of a Cell Lab Quanta™ SC flow cytometer (Beckman Coulter, Fullerton, CA, U.S.A.).

### Immunodetection of Carbonylated Proteins

Protein carbonylation was analyzed by the OxyBlot™ Protein Oxidation Detection Kit (Chemicon International, Billerica, MA, U.S.A.) following the manufacturer's specifications. Protein samples containing 30 µg of proteins were added to an equal volume of 12% SDS. Then protein carbonyl groups were derivatized to 2, 4-dinitrophenylhydrazone (DNP) by incubation with one additional volume of 2,4-dinitrophenylhydrazine for 15–20 min at room temperature. The derivatization reaction was stopped by the addition of neutralization solution. Proteins were separated by 12% SDS-PAGE and transferred to PVDF membrane (Millipore Corp., Billerica, MA, U.S.A.) using a TE 77 semidry transfer unit (GE Healthcare Bio-Sciences AB, Uppsala, Sweden). The oxidatively modified proteins were detected using anti-DNP antibodies (anti-dinitrophenyl-group antibodies) and visualized by a chemiluminescence detection kit (SuperSignal, Pierce Biotechnology, Rockford, IL, U.S.A.). To monitor the equal loading of samples, CBB R-250 was used to stain the proteins in a duplicate gel.

### Analysis of Protein Aggregation

Protein aggregation was determined as described by Li et al. [61]. Spores at an identical density were resuspended in extracting buffer containing 50 mM potassium phosphate buffer, pH 7.0, 1 mM EDTA, 5% glycerol, and 1 mM PMSF. Spores were broken by sonication on ice and the fungal debris was removed by centrifugation at 5,000×g for 30 min. The supernatant was decanted and centrifuged for 30 min at 15,000×g at 4°C. The pellets, which contained the membrane and aggregated proteins, were suspended in extracting buffer with brief sonication and centrifuged at 15,000×g for 30 min at 4°C. The pellets were resuspended again in extracting buffer, and then Nonidet P-40 was added at a final concentration of 2% v/v to solubilize membrane proteins. The mixture was centrifuged at 4°C for 30 min at 15,000×g to precipitate the Nonidet P-40-insoluble aggregrated proteins. The resultant protein pellets were washed again with one volume extracting buffer and finally dissolved in the same volume of lysis buffer containing 7 M urea, 2 M thiourea, 4% w/v CHAPS, 1% w/v dithiothreitol, and 2% v/v carrier ampholytes (pH 3–10). The loaded amount of aggregated protein in different treatments for gel analysis was calculated according to the ratio of corresponding protein concentrations of aggregated protein to total soluble protein.

### Measurement of ATP Contents

For ATP assay, spores were extracted with 2.5% TCA for 3 h at 4°C [61]. The homogenates were centrifuged at 10,000×g for 15 min. Ten microliters of supernatant was diluted with 115 µl of ATP-free H_2_O and 125 µl of ATP-free Tris-Acetate buffer (40 mM, pH 8.0; freshly prepared). ATP contents were determined with a luciferin/luciferase kit (ENLITEN® ATP Assay System, Promega, Madison, U.S.A.) following the manufacturer's specifications. The light emission by the reaction was detected with a Veritas Microplate Luminometer (Turner BioSystems, Sunnyvale, CA).

## Supporting Information

Figure S1 **High-purity mitochondria separations from fungal pathogen through a two-step Percoll density gradient.** (A) The first-step Percoll gradient for separation of fungal mitochondria. Crude mitochondrial pellet was laid on top of a step Percoll gradient consisting of 18% Percoll : 25% Percoll : 40% Percoll at 1∶4∶2 ratio. Mitochondrial bands were found at the 25%∶40% interface after centrifugation. (B) The second-step Percoll gradient for separation of fungal mitochondria. The mitochondria layer in the first-step Percoll gradient was aspirated and loaded onto a Percoll gradient containing 28% (v/v) Percoll. Highly purified mitochondria fractions, which were free of contamination, were enriched near the bottom of the gradient.
(DOC)

Figure S2 **Two-dimensional protein pattern revealed the changes of mitochondrial proteins in response to H_2_O_2_.** The fungal spores were treated with 0 mM (control) or 30 mM H_2_O_2_ for 60 min at 25°C after the spores were germinated in potato dextrose broth medium. Mitochondria were purified from the fungi through a two-step Percoll density gradient centrifugation as described under “Materials and Methods”. Mitochondrial proteins (500 µg) were separated by two-dimensional gel electrophoresis using 13 cm Immobiline Drystrip with a pH 3–10 nonlinear gradient. After electrophoresis, proteins were visualized by Coomassie Blue staining. Numbers indicate proteins that were differentially expressed under H_2_O_2_ stress and subsequently identified by mass spectrometry (listed in Table S3).
(DOC)

Figure S3 **Annotated spectra for identifications based on single peptides.** Nearly complete Y-ion series and partial complementary B-ion series were present as determined by manual inspection when proteins were identified with only one matching peptide or by multiple peptides with each ion scored below the threshold.
(DOC)

Table S1 **Identification of total cellular proteins of** ***P. expansum*** **upon exposure to H_2_O_2_ using ESI-Q-TOF or MALDI-TOF/TOF MS/MS.**
(DOC)

Table S2 **Prediction of subcellular location of proteins from total cellular extracts using the intracellular targeting prediction programs.** Three targeting prediction programs were used including TargetP (http://www.cbs.dtu.dk/services/TargetP/), Psort (http://psort.ims.u-tokyo.ac.jp/), and MitoProt (http://ihg2.helmholtz-muenchen.de/ihg/mitoprot.html).
(DOC)

Table S3 **Identification of mitochondrial proteins of** ***P. expansum*** **upon exposure to H_2_O_2_ using ESI-Q-TOF or MALDI-TOF/TOF MS/MS.**
(DOC)

Table S4 **Prediction of subcellular location of proteins from mitochondrial extracts using the intracellular targeting prediction programs.** Three targeting prediction programs were used including TargetP (http://www.cbs.dtu.dk/services/TargetP/), Psort (http://psort.ims.u-tokyo.ac.jp/), and MitoProt (http://ihg2.helmholtz-muenchen.de/ihg/mitoprot.html).
(DOC)

Table S5 **Scores and matched peptides of the identified proteins based on tandem mass spectrometry.** The detailed information of Mascot searching including the information of the mass spectra matching, the individual ions scores, and the sequences are given.
(XLS)
