# Supplementary material for: Hydrogen Peroxide Acts on Sensitive Mitochondrial Proteins to Induce Death of a Fungal Pathogen Revealed by Proteomic Analysis
Source: PLoS One. 2011 Jul 6;6(7):e21945. doi: 10.1371/journal.pone.0021945 (PMC3130790; doi:10.1371/journal.pone.0021945)
Supplement: Figure S1 — High-purity mitochondria separations from fungal pathogen through a two-step Percoll density gradient. (A) The first-step Percoll gradient for separation of fungal mitochondria. Crude mitochondrial pellet was laid on top of a step Percoll gradient consisting of 18% Percoll : 25% Percoll : 40% Percoll at 1∶4∶2 ratio. Mitochondrial bands were found at the 25%∶40% interface after centrifugation. (B) The second-step Percoll gradient for separation of fungal mitochondria. The mitochondria layer in the first-step Percoll gradient was aspirated and loaded onto a Percoll gradient containing 28% (v/v) Percoll. Highly purified mitochondria fractions, which were free of contamination, were enriched near the bottom of the gradient. (DOC) [file pone.0021945.s001.doc]

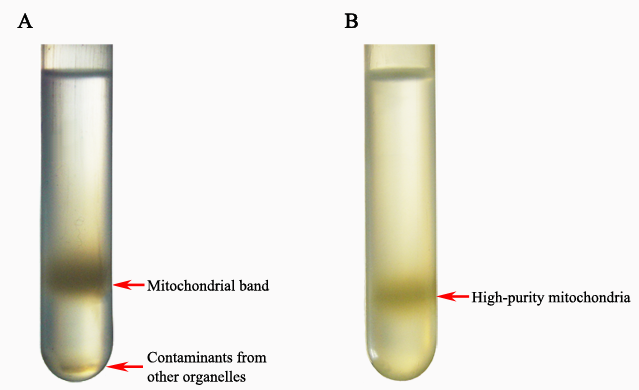


**Supporting Figure S1. High-purity mitochondria separations from fungal pathogen through a two-step Percoll density gradient.** (A) The first-step Percoll gradient for separation of fungal mitochondria. Crude mitochondrial pellet was laid on top of a step Percoll gradient consisting of 18% Percoll:25%Percoll:40% Percoll at 1:4:2 ratio. Mitochondrial bands were found at the 25%:40% interface after centrifugation. (B) The second-step Percoll gradient for separation of fungal mitochondria. The mitochondria layer in the first-step Percoll gradient was aspirated and loaded onto a Percoll gradient containing 28% (v/v) Percoll. Highly purified mitochondria fractions, which were free of contamination, were enriched near the bottom of the gradient.
