# Supplementary material for: Hydrogen Peroxide Acts on Sensitive Mitochondrial Proteins to Induce Death of a Fungal Pathogen Revealed by Proteomic Analysis
Source: PLoS One. 2011 Jul 6;6(7):e21945. doi: 10.1371/journal.pone.0021945 (PMC3130790; doi:10.1371/journal.pone.0021945)
Supplement: Figure S2 — Two-dimensional protein pattern revealed the changes of mitochondrial proteins in response to H2O2. The fungal spores were treated with 0 mM (control) or 30 mM H2O2 for 60 min at 25°C after the spores were germinated in potato dextrose broth medium. Mitochondria were purified from the fungi through a two-step Percoll density gradient centrifugation as described under “Materials and Methods”. Mitochondrial proteins (500 µg) were separated by two-dimensional gel electrophoresis using 13 cm Immobiline Drystrip with a pH 3–10 nonlinear gradient. After electrophoresis, proteins were visualized by Coomassie Blue staining. Numbers indicate proteins that were differentially expressed under H2O2 stress and subsequently identified by mass spectrometry (listed in Table S3). (DOC) [file pone.0021945.s002.doc]

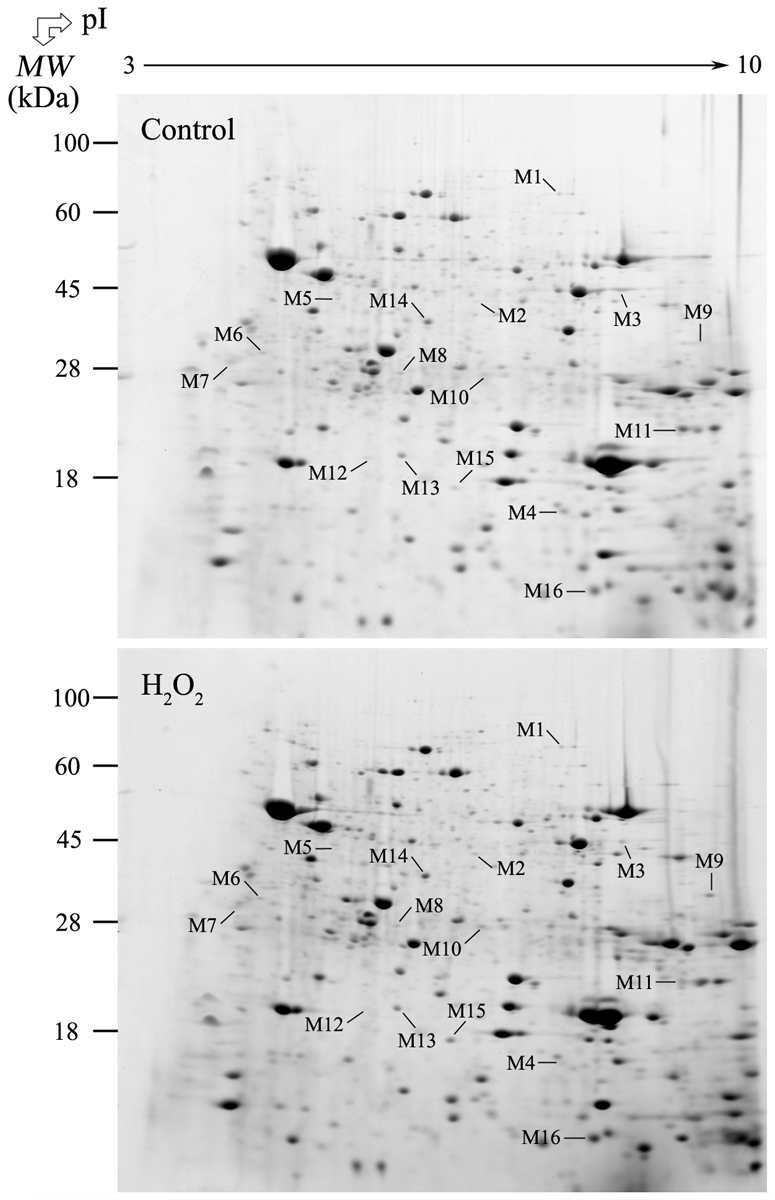


**Supporting Figure S2. Two-dimensional protein pattern revealed the changes of mitochondrial proteins in response to H_2_O_2_.** The fungal spores were treated with 0 mM (control) or 30 mM H_2_O_2_ for 60 min at 25 °C after the spores were germinated in potato dextrose broth medium. Mitochondria were purified from the fungi through a two-step Percoll density gradient centrifugation as described under “Materials and Methods”. Mitochondrial proteins (500 μg) were separated by two-dimensional gel electrophoresis using 13 cm Immobiline Drystrip with a pH 3–10 nonlinear gradient. After electrophoresis, proteins were visualized by Coomassie Blue staining. Numbers indicate proteins that were differentially expressed under H_2_O_2_ stress and subsequently identified by mass spectrometry (listed in Table S3).
