# Supplementary material for: Hydrogen Peroxide Acts on Sensitive Mitochondrial Proteins to Induce Death of a Fungal Pathogen Revealed by Proteomic Analysis
Source: PLoS One. 2011 Jul 6;6(7):e21945. doi: 10.1371/journal.pone.0021945 (PMC3130790; doi:10.1371/journal.pone.0021945)
Supplement: Figure S3 — Annotated spectra for identifications based on single peptides. Nearly complete Y-ion series and partial complementary B-ion series were present as determined by manual inspection when proteins were identified with only one matching peptide or by multiple peptides with each ion scored below the threshold. (DOC) [file pone.0021945.s003.doc]

**Supporting Figure S3**

**Annotated spectra for identifications based on single peptides**

Spot numbers were consistent with those in 2-DE gel

**Spot Number: C3**

**MS/MS Peptide Sequences: DQGLNVIVGVR**

**m/z: 1169.691500**

**Charge: 1+**

**Mr (experimental): 1168.6842**

**Mr (predicted): 1168.6564**

**Mass error: 0.0278 Da**

**Mascot Ions Score: 27**

**Expect Value: 2.5**

**
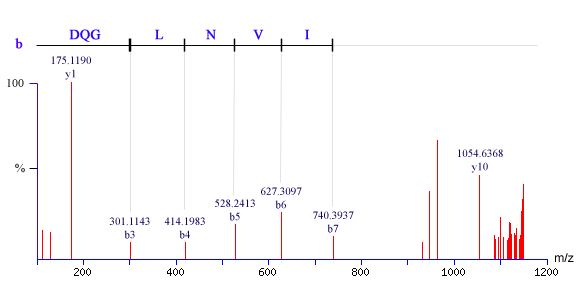
**

**Spot Number: C28**

**MS/MS Peptide Sequences: DALNEALAEELER**

**m/z: 1472.8010**

**Charge: 1+**

**Mr (experimental): 1471.7937**

**Mr (predicted): 1471.7154**

**Mass error: 0.0783 Da**

**Mascot Ions Score: 63**

**Expect Value: 0.00061**

**
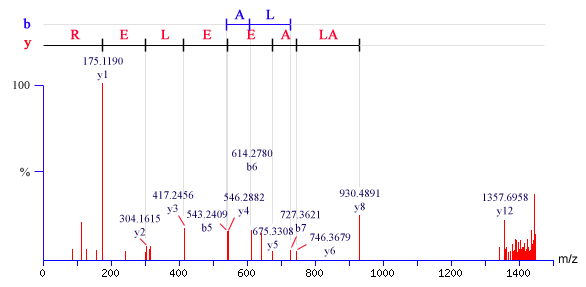
**

**Spot Number: C1**

**MS/MS Peptide Sequences: ISPMYPFIDGEVR**

**m/z: 1523.7798**

**Charge: 1+**

**Mr (experimental): 1522.7725**

**Mr (predicted): 1522.7490**

**Mass error: 0.0235 Da**

**Mascot Ions Score: 35**

**Expect Value: 0.37**

**
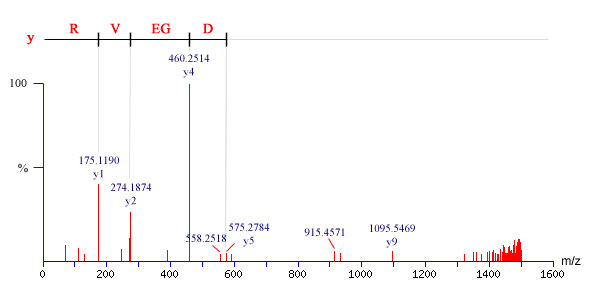
**

**Spot Number: C11**

**MS/MS Peptide Sequences: YNLIILSDEVYDR**

**m/z: 1612.8313**

**Charge: 1+**

**Mr (experimental): 1611.8240**

**Mr (predicted): 1611.8144**

**Mass error: 0.0096 Da**

**Mascot Ions Score: 69**

**Expect Value: 0.00013**

**
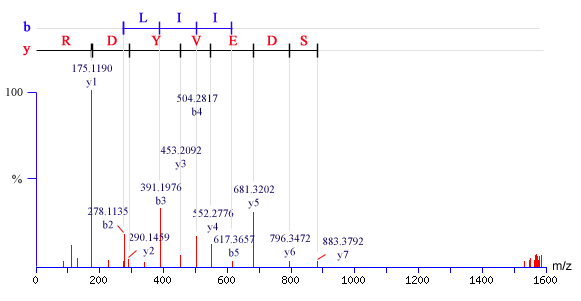
**

**Spot Number: C2**

**MS/MS Peptide Sequences: GVAAEDTGAPITIPVGPSTLGR**

**m/z: 2063.1692**

**Charge: 1+**

**Mr (experimental): 2062.1619**

**Mr (predicted): 2062.0695**

**Mass error: 0.0924 Da**

**Mascot Ions Score: 67**

**Expect Value: 0.00024**

**
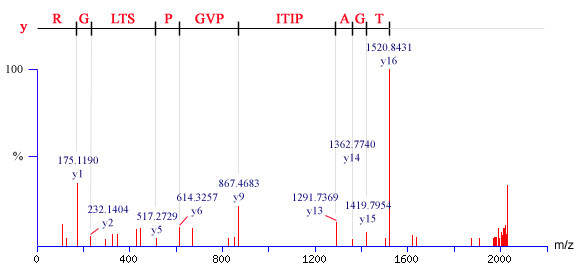
**

**Spot Number: C19**

**MS/MS Peptide Sequences: VGAFHYCTNR**

**m/z: 1224.5892**

**Charge: 1+**

**Mr (experimental): 1223.5819**

**Mr (predicted): 1223.5506**

**Mass error: 0.0313 Da**

**Mascot Ions Score: 35**

**Expect Value: 0.43**

**
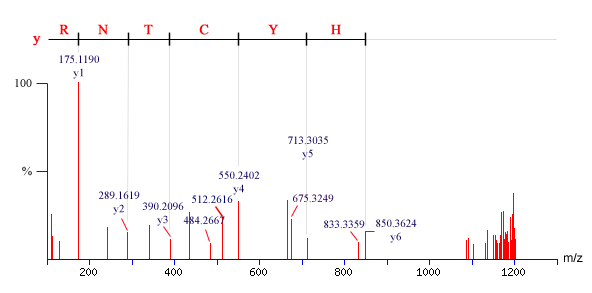
**

**Spot Number: C15**

**MS/MS Peptide Sequences: GTSGFAIDVLAR**

**m/z: 1206.6838**

**Charge: 1+**

**Mr (experimental): 1205.6765**

**Mr (predicted): 1205.6404**

**Mass error: 0.0361 Da**

**Mascot Ions Score: 26**

**Expect Value: 3.1**

**
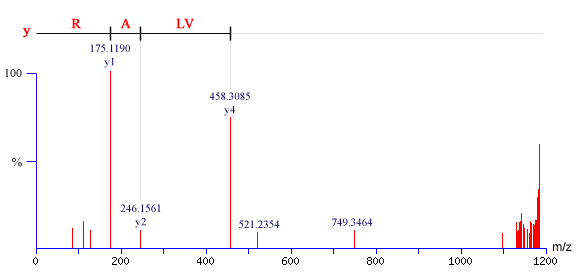
**

**Spot Number: C24**

**MS/MS Peptide Sequences: VSLIHDGLAR**

**m/z: 1080.6248**

**Charge: 1+**

**Mr (experimental): 1079.6175**

**Mr (predicted): 1079.6087**

**Mass error: 0.0088 Da**

**Mascot Ions Score: 66**

**Expect Value: 0.00031**

**
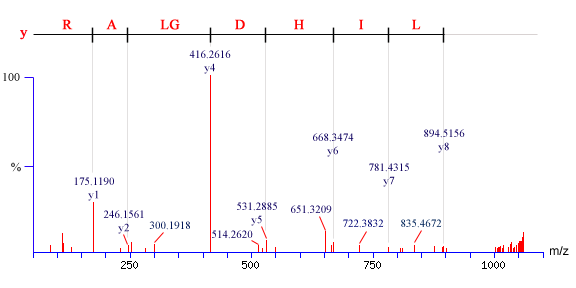
**

**Spot Number: C4**

**MS/MS Peptide Sequences: LFILDEADEMLSR**

**m/z: 1551.8145**

**Charge: 1+**

**Mr (experimental): 1550.8072**

**Mr (predicted): 1550.7650**

**Mass error: 0.0422 Da**

**Mascot Ions Score: 52**

**Expect Value: 0.0062**

**
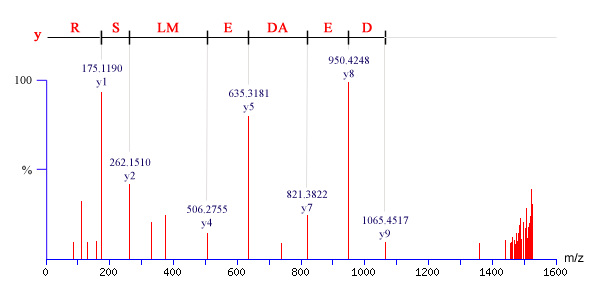
**

**Spot Number: C10**

**MS/MS Peptide Sequences: AIYALSSAVR**

**m/z: 1050.5830**

**Charge: 1+**

**Mr (experimental): 1049.5757**

**Mr (predicted): 1049.5869**

**Mass error: -0.0112 Da**

**Mascot Ions Score: 60**

**Expect Value: 0.0014**

**
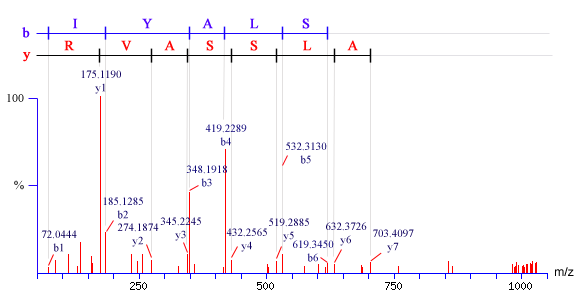
**

**Spot Number: M8**

**MS/MS Peptide Sequences: IPAPGPLSGR**

**m/z: 482.7541**

**Charge: 2+**

**Mr (experimental): 963.4936**

**Mr (predicted): 963.5502**

**Mass error: -0.0565 Da**

**Mascot Ions Score: 54**

**Expect Value: 0.0064**

**
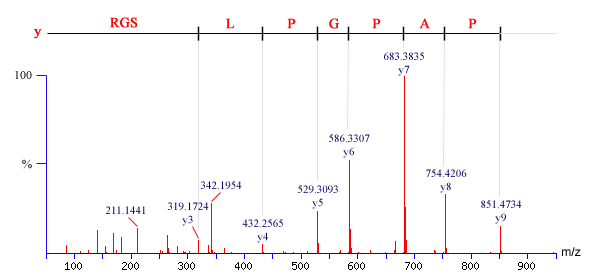
**

**Spot Number: M15**

**MS/MS Peptide Sequences: NLFEDIYVR**

**m/z: 1168.5991**

**Charge: 1+**

**Mr (experimental): 1167.5918**

**Mr (predicted): 1167.5924**

**Mass error: -0.0006 Da**

**Mascot Ions Score: 47**

**Expect Value: 0.025**

**
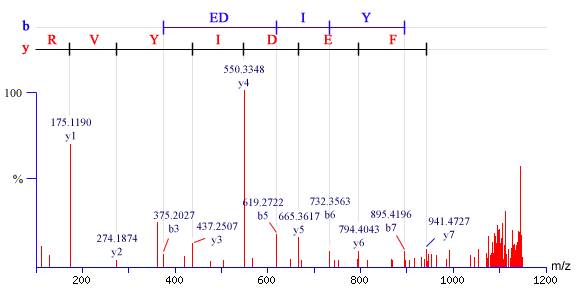
**

**Spot Number: M2**

**MS/MS Peptide Sequences: LLGTTENELGLR**

**m/z: 658.3830**

**Charge: 2+**

**Mr (experimental): 1314.7514**

**Mr (predicted): 1314.7143**

**Mass error: 0.0371 Da**

**Mascot Ions Score: 71**

**Expect Value: 0.0001**

**
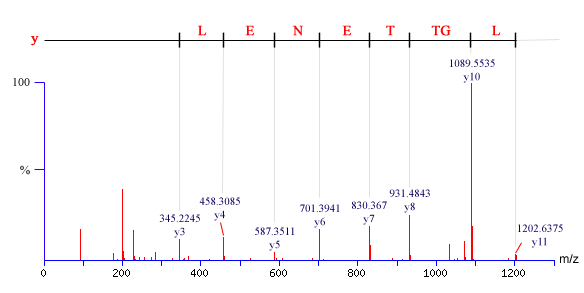
**

**Spot Number: M5**

**MS/MS Peptide Sequences: LHATFDESAR**

**m/z: 1146.5475**

**Charge: 1+**

**Mr (experimental): 1145.5402**

**Mr (predicted): 1145.5465**

**Mass error: -0.0063 Da**

**Mascot Ions Score: 46**

**Expect Value: 0.034**

**
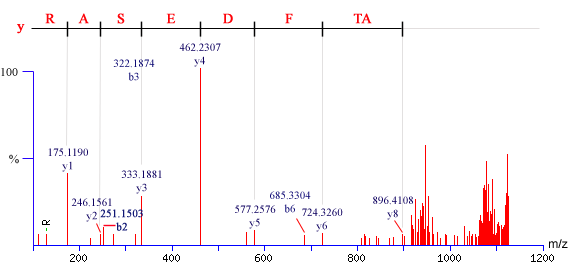
**
