# Supplementary material for: Hydrogen Peroxide Acts on Sensitive Mitochondrial Proteins to Induce Death of a Fungal Pathogen Revealed by Proteomic Analysis
Source: PLoS One. 2011 Jul 6;6(7):e21945. doi: 10.1371/journal.pone.0021945 (PMC3130790; doi:10.1371/journal.pone.0021945)
Supplement: Table S1 — Identification of total cellular proteins of P. expansum upon exposure to H2O2 using ESI-Q-TOF or MALDI-TOF/TOF MS/MS. (DOC) [file pone.0021945.s004.doc]

***Supporting Table S1.*** *Identification of total cellular proteins of P. expansum upon exposure to H_2_O_2_ using ESI-Q-TOF or MALDI-TOF/TOF MS/MS.*

| **Spot^a^** | **ORF name^b^** | **Accession number^c^** | **Protein function^d^** | **Theo. *Mr* (kDa)/ pI^e^** | **Expt. *Mr* (kDa)/pI^f^** | **Species^g^** | **Mascot**  **Score^h^** | **NP^i^** | **SC**  **(%)^j^** | **Ratio^k^** | **Method^l^** |
| --- | --- | --- | --- | --- | --- | --- | --- | --- | --- | --- | --- |
| **Putative Mitochondrial Proteins** | | | | | | | | | | | |
| C26 | Pc20g01610 | gi|255943905 | malate dehydrogenase | 35.82/8.44 | 32.2/5.6 | *Penicillium chrysogenum* | 219 | 4 | 14 | +2.28 | M-TOF/TOF |
| C3 | Pc13g15810 | gi|255938411 | ketol-acid reductoisomerase | 44.15/8.79 | 45.5/6.3 | *Penicillium chrysogenum* | 64 | 3 | 9 | -2.63 | M-TOF/TOF |
| C5 | Pc22g10140 | gi|255948510 | acetyl-CoA C-acetyltransferase | 41.17/5.93 | 58.5/7.0 | *Penicillium chrysogenum* | 330 | 7 | 27 | -2.01 | M-TOF/TOF |
| C28 | ― | gi|259488245 | pyruvate dehydrogenase E1 component, beta subunit | 40.89/6.85 | 58.1/6.6 | *Aspergillus nidulans* | 63 | 1 | 3 | -1.75 | M-TOF/TOF |
| C1 | Pc16g01790 | gi|255939568 | glycerol-3-phosphate dehydrogenase | 76.91/6.24 | 44.5/5.7 | *Penicillium chrysogenum* | 87 | 3 | 5 | -1.62 | M-TOF/TOF |
| C7 | Pc21g08400 | gi|255954253 | mitochondrial 3-hydroxyisobutyryl-CoA hydrolase, putative | 53.02/7.79 | 32.2/6.4 | *Penicillium chrysogenum* | 164 | 5 | 12 | -1.96 | M-TOF/TOF |
| C11 | Pc22g06360 | gi|255947798 | mitochondrial kynurenine aminotransferase KAT | 52.48/5.94 | 44.7/5.4 | *Penicillium chrysogenum* | 141 | 4 | 11 | -1.71 | M-TOF/TOF |
| C21 | Pc22g19990 | gi|255950268 | molecular chaperone DnaK | 72.60/5.58 | 73.1/5.5 | *Penicillium chrysogenum* | 574 | 17 | 26 | +1.63 | ESI-Q-TOF |
| C23 | Pc22g19990 | gi|255950268 | molecular chaperone DnaK | 72.60/5.58 | 70.5/5.2 | *Penicillium chrysogenum* | 408 | 5 | 10 | +1.68 | M-TOF/TOF |
| C2 | Pc21g10070 | gi|255954579 | F-type H+-transporting ATPase subunit beta | 55.24/5.25 | 44.5/6.1 | *Penicillium chrysogenum* | 49 | 2 | 7 | -1.69 | M-TOF/TOF |
| **Other Proteins** | | | | | | | | | | | |
| C8 | Pc21g14560 | gi|255955435 | glyceraldehyde 3-phosphate dehydrogenase | 36.15/6.23 | 25.0/4.6 | *Penicillium chrysogenum* | 260 | 7 | 28 | -1.85 | M-TOF/TOF |
| C9 | Pc21g14560 | gi|255955435 | glyceraldehyde 3-phosphate dehydrogenase | 36.15/6.23 | 48.9/5.6 | *Penicillium chrysogenum* | 184 | 7 | 27 | +1.76 | M-TOF/TOF |
| C17 | Pc22g23830 | gi|255951014 | aspartate aminotransferase | 46.89/6.17 | 45.1/6.3 | *Penicillium chrysogenum* | 293 | 11 | 26 | -1.57 | ESI-Q-TOF |
| C19 | Pc22g23830 | gi|255951014 | aspartate aminotransferase | 46.89/6.17 | 44.5/6.8 | *Penicillium chrysogenum* | 85 | 3 | 7 | -1.56 | M-TOF/TOF |
| C13 | ― | gi|238504174 | glutamine synthetase | 42.48/5.79 | 74.0/5.7 | *Aspergillus flavus* | 274 | 7 | 18 | -1.66 | M-TOF/TOF |
| C6 | Pc21g11360 | gi|255954831 | glutamine amidotransferase | 60.15/5.41 | 32.1/6.5 | *Penicillium chrysogenum* | 228 | 6 | 13 | -1.99 | M-TOF/TOF |
| C14 | Pc14g02010 | gi|255938850 | chorismate synthase | 44.21/6.23 | 49.3/6.8 | *Penicillium chrysogenum* | 385 | 17 | 41 | -1.64 | ESI-Q-TOF |
| C12 | ― | gi|255940110 | phosphoglycerate kinase pgkA | 44.11/5.98 | 85.0/5.4 | *Penicillium chrysogenum* | 143 | 4 | 14 | +1.70 | M-TOF/TOF |
| C20 | Pc16g13350 | gi|255941730 | 3-isopropylmalate dehydrogenase | 39.18/5.21 | 42.5/4.7 | *Penicillium chrysogenum* | 252 | 5 | 16 | -1.68 | M-TOF/TOF |
| C22 | Pc13g09680 | gi|255936729 | aspartyl proteinase candidapepsin | 43.59/5.08 | 17.8/4.8 | *Penicillium chrysogenum* | 203 | 4 | 13 | -1.69 | M-TOF/TOF |
| C15 | Pc22g15910 | gi|255949480 | X-Pro aminopeptidase | 67.99/5.47 | 32.1/6.7 | *Penicillium chrysogenum* | 48 | 3 | 4 | -1.62 | M-TOF/TOF |
| C16 | Pc18g02330 | gi|255942617 | small subunit ribosomal protein S12e | 16.47/4.86 | 17.9/4.9 | *Penicillium chrysogenum* | 540 | 11 | 62 | -1.62 | ESI-Q-TOF |
| C18 | Pc18g02330 | gi|255942617 | small subunit ribosomal protein S12e | 16.47/4.86 | 44.1/5.5 | *Penicillium chrysogenum* | 148 | 3 | 21 | -1.78 | M-TOF/TOF |
| C24 | Pc18g02330 | gi|255942617 | small subunit ribosomal protein S12e | 16.47/4.86 | 32.2/6.2 | *Penicillium chrysogenum* | 66 | 1 | 6 | +1.79 | M-TOF/TOF |
| C25 | Pc22g21650 | gi|255950588 | tubulin alpha | 50.99/4.91 | 85.0/5.5 | *Penicillium chrysogenum* | 220 | 5 | 14 | +1.98 | M-TOF/TOF |
| C4 | Pc20g06740 | gi|255944863 | translation initiation factor eIF-4A | 44.66/5.02 | 58.5/5.9 | *Penicillium chrysogenum* | 72 | 2 | 6 | -2.04 | M-TOF/TOF |
| C10 | ― | gi|116198003 | hypothetical protein | 28.50/4.77 | 49.1/6.2 | *Chaetomium globosum* | 60 | 1 | 3 | -1.73 | M-TOF/TOF |
| C27 | ― | ― | ND^m^ | ― | 72.5/5.1 | ― | ― | ― | ― | +2.10 | M-TOF/TOF |

^a^Spot number corresponding to spots in Figure 2A.

^b^The corresponding open reading frame of a matched protein.

^c^Accession number from NCBI database of a matched protein.

^d^Protein function defined by the KEGG database.

^e^Theoretical molecular mass and isoelectric point based on amino acid sequence of the identified protein.

^f^Experimental molecular mass and isoelectric point estimated from the 2D gels.

^g^Species of the matched protein.

^h^Score obtained from Mascot for each match. Mascot scores greater than 44 are statistically significant (*p* < 0.05).

^i^Number of unique peptides identified.

^j^Amino acid sequence coverage for the identified proteins.

^k^The average fold change of protein expression levels in H_2_O_2_-treated fungi versus control from three replicate 2D gels obtained from independent protein extraction. The sign + represents over-expressed proteins and - represents down-regulated proteins.

^l^Approach for MS/MS analysis. M-TOF/TOF, MALDI-TOF/TOF tandem mass spectrometry. ESI-Q-TOF, ESI-Q-TOF tandem mass spectrometry.

^m^ No match with database.
