# Supplementary material for: Hydrogen Peroxide Acts on Sensitive Mitochondrial Proteins to Induce Death of a Fungal Pathogen Revealed by Proteomic Analysis
Source: PLoS One. 2011 Jul 6;6(7):e21945. doi: 10.1371/journal.pone.0021945 (PMC3130790; doi:10.1371/journal.pone.0021945)
Supplement: Table S2 — Prediction of subcellular location of proteins from total cellular extracts using the intracellular targeting prediction programs. Three targeting prediction programs were used including TargetP (http://www.cbs.dtu.dk/services/TargetP/), Psort (http://psort.ims.u-tokyo.ac.jp/), and MitoProt (http://ihg2.helmholtz-muenchen.de/ihg/mitoprot.html). (DOC) [file pone.0021945.s005.doc]

**Supporting Table S2.** Prediction of subcellular location of proteins from total cellular extracts using the intracellular targeting prediction programs.

| **Spot*^a^*** | **ORF name** | **Accession number** | **Protein function** | **TargetP*^b^*** | **MitoProt*^c^*** | **Psort*^d^*** |
| --- | --- | --- | --- | --- | --- | --- |
| **Putatively Mitochondrial Proteins** | | | | | | |
| C26 | Pc20g01610 | gi|255943905 | malate dehydrogenase | M2 | 0.9856 | M (0.593) |
| C3 | Pc13g15810 | gi|255938411 | ketol-acid reductoisomerase | M1 | 0.9983 | M (0.889) |
| C5 | Pc22g10140 | gi|255948510 | acetyl-CoA C-acetyltransferase | M4 | 0.5932 | M (0.370) |
| C28 | ― | gi|259488245 | pyruvate dehydrogenase E1 component, beta subunit | M1 | 0.9993 | M (0.981) |
| C1 | Pc16g01790 | gi|255939568 | glycerol-3-phosphate dehydrogenase | M2 | 0.9944 | M (0.704) |
| C7 | Pc21g08400 | gi|255954253 | mitochondrial 3-hydroxyisobutyryl-CoA hydrolase, putative | M5 | 0.7393 | M (0.667) |
| C11 | Pc22g06360 | gi|255947798 | mitochondrial kynurenine aminotransferase KAT | M2 | 0.9292 | M (0.926) |
| C21 | Pc22g19990 | gi|255950268 | molecular chaperone DnaK | M1 | 0.9955 | M (0.926) |
| C23 | Pc22g19990 | gi|255950268 | molecular chaperone DnaK | M1 | 0.9955 | M (0.926) |
| C2 | Pc21g10070 | gi|255954579 | F-type H+-transporting ATPase subunit beta | M1 | 0.9951 | M (0.926) |
| **Other Proteins** | | | | | | |
| C8 | Pc21g14560 | gi|255955435 | glyceraldehyde 3-phosphate dehydrogenase | M4 | 0.8439 | C (1.000) |
| C9 | Pc21g14560 | gi|255955435 | glyceraldehyde 3-phosphate dehydrogenase | M4 | 0.8439 | C (1.000) |
| C17 | Pc22g23830 | gi|255951014 | aspartate aminotransferase | 2 | 0.0655 | C (0.278) |
| C19 | Pc22g23830 | gi|255951014 | aspartate aminotransferase | 2 | 0.0655 | C (0.278) |
| C13 | ― | gi|238504174 | glutamine synthetase | 1 | 0.0220 | C (0.352) |
| C6 | Pc21g11360 | gi|255954831 | glutamine amidotransferase | 2 | 0.6449 | C (0.833) |
| C14 | Pc14g02010 | gi|255938850 | chorismate synthase | 4 | 0.0144 | C (0.463) |
| C12 | ― | gi|255940110 | phosphoglycerate kinase pgkA | 2 | 0.1984 | C (1.000) |
| C20 | Pc16g13350 | gi|255941730 | 3-isopropylmalate dehydrogenase | 2 | 0.0118 | C (0.481) |
| C22 | Pc13g09680 | gi|255936729 | aspartyl proteinase candidapepsin | S2 | 0.0535 | Ex (0.889) |
| C15 | Pc22g15910 | gi|255949480 | X-Pro aminopeptidase | 2 | 0.0403 | C (0.722) |
| C16 | Pc18g02330 | gi|255942617 | small subunit ribosomal protein S12e | 1 | 0.0044 | C (0.537) |
| C18 | Pc18g02330 | gi|255942617 | small subunit ribosomal protein S12e | 1 | 0.0044 | C (0.537) |
| C24 | Pc18g02330 | gi|255942617 | small subunit ribosomal protein S12e | 1 | 0.0044 | C (0.537) |
| C25 | Pc22g21650 | gi|255950588 | tubulin alpha | 2 | 0.0693 | C (0.407) |
| C4 | Pc20g06740 | gi|255944863 | translation initiation factor eIF-4A | 1 | 0.0326 | N (0.500) |
| C10 | ― | gi|116198003 | hypothetical protein | 2 | 0.3011 | N (0.648) |

*^a^*Spot number corresponding to spots in Figure 2A.

*^b^*TargetP, predicted localization of sequence by TargetP; M1–5, mitochondrial; S1–5, secretory pathway; 1–5, other (1, high probability; 5, low probability).

*^c^*MitoProt, probability of mitochondrial targeting (*p* = 0–1).

*^d^*Psort, predicted localization by Psort; M, mitochondria; C, cytosol; Ex, secreted; N, nucleus (*p* = 0–1).
