# Supplementary material for: Hydrogen Peroxide Acts on Sensitive Mitochondrial Proteins to Induce Death of a Fungal Pathogen Revealed by Proteomic Analysis
Source: PLoS One. 2011 Jul 6;6(7):e21945. doi: 10.1371/journal.pone.0021945 (PMC3130790; doi:10.1371/journal.pone.0021945)
Supplement: Table S3 — Identification of mitochondrial proteins of P. expansum upon exposure to H2O2 using ESI-Q-TOF or MALDI-TOF/TOF MS/MS. (DOC) [file pone.0021945.s006.doc]

***Supporting Table S3.*** *Identification of mitochondrial proteins of P. expansum upon exposure to H_2_O_2_ using ESI-Q-TOF or MALDI-TOF/TOF MS/MS.*

| **Spot^a^** | **ORF name^b^** | **Accession number^c^** | **Protein function^d^** | **Theo. *Mr* (kDa)/ pI^e^** | **Expt. *Mr* (kDa)/pI^f^** | **Species^g^** | **Mascot**  **Score^h^** | **NP^i^** | **SC**  **(%)^j^** | **Ratio^k^** | **Method^l^** |
| --- | --- | --- | --- | --- | --- | --- | --- | --- | --- | --- | --- |
| **Electron transport chain** | | | | | | | | | | | |
| M4 | Pc22g06720 | gi|255947866 | NADH dehydrogenase (ubiquinone) flavoprotein 1 | 54.89/8.38 | 16.6/7.5 | *Penicillium chrysogenum* | 247 | 7 | 14 | -1.71 | ESI-Q-TOF |
| M8 | Pc16g10510 | gi|255941184 | NADH dehydrogenase (ubiquinone) flavoprotein 2 | 29.84/6.52 | 27.8/6.0 | *Penicillium chrysogenum* | 54 | 2 | 8 | -1.52 | ESI-Q-TOF |
| M16 | ― | gi|71002284 | NADH-ubiquinone oxidoreductase 12 kDa subunit | 12.28/7.74 | 12.3/7.8 | *Aspergillus fumigatus* | 71 | 3 | 29 | +1.74 | M-TOF/TOF |
| M3 | Pc12g05480 | gi|255931673 | ubiquinol-cytochrome *c* reductase core subunit 2 | 48.26/8.89 | 45.0/8.4 | *Penicillium chrysogenum* | 503 | 12 | 32 | -2.75 | ESI-Q-TOF |
| M11 | Pc12g03370 | gi|255931331 | mitochondrial F1-ATPase alpha-subunit Atp1 | 59.78/9.00 | 24.1/9.1 | *Penicillium chrysogenum* | 220 | 5 | 10 | -1.81 | ESI-Q-TOF |
| M7 | Pc21g10070 | gi|255954579 | F-type H+-transporting ATPase subunit beta | 55.24/5.25 | 29.6/4.3 | *Penicillium chrysogenum* | 120 | 4 | 12 | -2.32 | M-TOF/TOF |
| M6 | Pc21g10070 | gi|255954579 | F-type H+-transporting ATPase subunit beta | 55.24/5.25 | 31.5/4.8 | *Penicillium chrysogenum* | 563 | 10 | 24 | -3.34 | ESI-Q-TOF |
| M14 | Pc13g03260 | gi|255935475 | F-type H+-transporting ATPase subunit gamma | 32.42/6.04 | 36.5/6.2 | *Penicillium chrysogenum* | 138 | 5 | 16 | -1.61 | ESI-Q-TOF |
| **Proteins involved in the tricarboxylic cycle and general metabolism** | | | | | | | | | | | |
| M13 | Pc22g11710 | gi|255948700 | pyruvate dehydrogenase E1 component subunit alpha | 45.38/8.49 | 21.5/6.0 | *Penicillium chrysogenum* | 292 | 7 | 17 | -1.9 | ESI-Q-TOF |
| M15 | ― | gi|121702397 | pyruvate dehydrogenase E1 component alpha subunit, putative | 45.42/7.67 | 20.5/6.3 | *Aspergillus clavatus* | 47 | 1 | 2 | +1.68 | M-TOF/TOF |
| M2 | Pc12g04310 | gi|255931489 | NAD-dependent formate dehydrogenase | 50.35/6.25 | 43.8/6.6 | *Penicillium chrysogenum* | 71 | 1 | 2 | +1.71 | ESI-Q-TOF |
| M1 | Pc16g01790 | gi|255939568 | glycerol-3-phosphate dehydrogenase | 76.92/6.24 | 77.1/7.4 | *Penicillium chrysogenum* | 413 | 10 | 17 | -1.87 | ESI-Q-TOF |
| **Membrane carriers** | | | | | | | | | | | |
| M10 | Pc13g12190 | gi|255937217 | mitochondrial import protein Metaxin | 33.04/6.14 | 27.2/6.7 | *Penicillium chrysogenum* | 131 | 3 | 12 | +1.53 | ESI-Q-TOF |
| M9 | Pc06g01110 | gi|255930321 | mitochondrial phosphate carrier protein (Mir1), putative | 32.22/9.44 | 32.5/9.4 | *Penicillium chrysogenum* | 217 | 7 | 25 | +1.61 | M-TOF/TOF |
| **Unknown function** | | | | | | | | | | | |
| M5 | Pc13g07690 | gi|255936343 | mitochondrial inner membrane protein | 71.92/6.10 | 44.1/5.4 | *Penicillium chrysogenum* | 137 | 5 | 8 | -2.50 | M-TOF/TOF |
| **Contaminants** | | | | | | | | | | | |
| M12 | Pc22g22060 | gi|255950668 | GTP-binding protein | 23.57/5.48 | 21.2/5.7 | *Penicillium chrysogenum* | 212 | 5 | 24 | -1.58 | ESI-Q-TOF |

^a^Spot number corresponding to spots in Figure S2.

^b^The corresponding open reading frame of a matched protein.

^c^Accession number from NCBI database of a matched protein.

^d^Protein function defined by the KEGG database.

^e^Theoretical molecular mass and isoelectric point based on amino acid sequence of the identified protein.

^f^Experimental molecular mass and isoelectric point estimated from the 2D gels.

^g^Species of the matched protein.

^h^Score obtained from Mascot for each match. Mascot scores greater than 44 are statistically significant (*p* < 0.05).

^i^Number of unique peptides identified.

^j^Amino acid sequence coverage for the identified proteins.

^k^The average fold change of protein expression levels in H_2_O_2_-treated fungi versus control from three replicate 2D gels obtained from independent mitochondrial extractions. The sign + represents over-expressed proteins and - represents down-regulated proteins.

^l^Approach for MS/MS analysis. M-TOF/TOF, MALDI-TOF/TOF tandem mass spectrometry. ESI-Q-TOF, ESI-Q-TOF tandem mass spectrometry.
