# Supplementary material for: Hydrogen Peroxide Acts on Sensitive Mitochondrial Proteins to Induce Death of a Fungal Pathogen Revealed by Proteomic Analysis
Source: PLoS One. 2011 Jul 6;6(7):e21945. doi: 10.1371/journal.pone.0021945 (PMC3130790; doi:10.1371/journal.pone.0021945)
Supplement: Table S4 — Prediction of subcellular location of proteins from mitochondrial extracts using the intracellular targeting prediction programs. Three targeting prediction programs were used including TargetP (http://www.cbs.dtu.dk/services/TargetP/), Psort (http://psort.ims.u-tokyo.ac.jp/), and MitoProt (http://ihg2.helmholtz-muenchen.de/ihg/mitoprot.html). (DOC) [file pone.0021945.s007.doc]

**Supporting Table S4.** Prediction of subcellular location of proteins from mitochondrial extracts using the intracellular targeting prediction programs.

| **Spot*^a^*** | **ORF name** | **Accession number** | **Protein function** | **TargetP*^b^*** | **MitoProt*^c^*** | **Psort*^d^*** |
| --- | --- | --- | --- | --- | --- | --- |
| **Electron transport chain** | | | | | | |
| M4 | Pc22g06720 | gi|255947866 | NADH dehydrogenase (ubiquinone) flavoprotein 1 | M1 | 0.9909 | M (0.870) |
| M8 | Pc16g10510 | gi|255941184 | NADH dehydrogenase (ubiquinone) flavoprotein 2 | M2 | 0.9902 | M (0.759) |
| M16 | ― | gi|71002284 | NADH-ubiquinone oxidoreductase 12 kda subunit | 2 | 0.0152 | M (0.444) |
| M3 | Pc12g05480 | gi|255931673 | ubiquinol-cytochrome c reductase core subunit 2 | M1 | 0.9980 | M (0.981) |
| M11 | Pc12g03370 | gi|255931331 | mitochondrial F1-ATPase alpha-subunit Atp1 | M1 | 0.9964 | M (0.981) |
| M7 | Pc21g10070 | gi|255954579 | F-type H+-transporting ATPase subunit beta | M1 | 0.9951 | M (0.926) |
| M6 | Pc21g10070 | gi|255954579 | F-type H+-transporting ATPase subunit beta | M1 | 0.9951 | M (0.926) |
| M14 | Pc13g03260 | gi|255935475 | F-type H+-transporting ATPase subunit gamma | M1 | 09847 | M (0.833) |
| **Proteins involved in the tricarboxylic cycle and general metabolism** | | | | | | |
| M13 | Pc22g11710 | gi|255948700 | pyruvate dehydrogenase E1 component subunit alpha | M1 | 0.9981 | M (0.981) |
| M15 | ― | gi|121702397 | pyruvate dehydrogenase E1 component alpha subunit, putative | M1 | 0.9870 | M (0.981) |
| M2 | Pc12g04310 | gi|255931489 | NAD-dependent formate dehydrogenase | 3 | 0.0565 | C (0.370) |
| M1 | Pc16g01790 | gi|255939568 | glycerol-3-phosphate dehydrogenase | M2 | 0.9944 | M (0.704) |
| **Membrane carriers** | | | | | | |
| M10 | Pc13g12190 | gi|255937217 | mitochondrial import protein Metaxin | 2 | 0.0248 | N (0.463) |
| M9 | Pc06g01110 | gi|255930321 | mitochondrial phosphate carrier protein (Mir1), putative | 3 | 0.1660 | Ex (0.296) |
| **Unknown function** | | | | | | |
| M5 | Pc13g07690 | gi|255936343 | mitochondrial inner membrane protein | M2 | 0.9965 | M (1.000) |
| **Contaminants** | | | | | | |
| M12 | Pc22g22060 | gi|255950668 | GTP-binding protein | 2 | 0.0509 | N (0.537) |

*^a^*Spot number corresponding to spots in Figure S2.

*^b^*TargetP, predicted localization of sequence by TargetP; M1–5, mitochondrial; 1–5, other (1, high probability; 5, low probability).

*^c^*MitoProt, probability of mitochondrial targeting (*p* = 0–1).

*^d^*Psort, predicted localization by Psort; M, mitochondria; C, cytosol; N, nucleus; Ex, secreted (*p* = 0–1).
